# Breakage-Fusion-Bridge Events Trigger Complex Genome Rearrangements and Amplifications in Developmentally Arrested T Cell Lymphomas

**DOI:** 10.1016/j.celrep.2019.05.014

**Published:** 2019-06-04

**Authors:** Joy J. Bianchi, Valentine Murigneux, Marie Bedora-Faure, Chloé Lescale, Ludovic Deriano

**Affiliations:** 1 Genome Integrity, Immunity and Cancer Unit, Equipe Labellisée Ligue Contre le Cancer, Department of Immunology, Department of Genomes and Genetics, Institut Pasteur, 75015 Paris, France; 2 Cellule Pasteur, University of Paris René Descartes, Sorbonne Paris Cité, 75015 Paris, France

**Keywords:** cancer genome landscape, T cell development, T cell lymphoma, DNA damage, structural variation, breakage-fusion-bridge, RAG1/2 nuclease

## Abstract

To reveal the relative contribution of the recombination activating gene (RAG)1/2 nuclease to lymphomagenesis, we conducted a genome-wide analysis of T cell lymphomas from p53-deficient mice expressing or lacking RAG2. We found that while *p53*^−/−^ lymphoblastic T cells harbor primarily ectopic DNA deletions, *Rag2*^*−/−*^*p53*^*−/−*^ T cell lymphomas display complex genomic rearrangements associated with amplification of the chromosomal location 9qA4-5.3. We show that this amplicon is generated by breakage-fusion-bridge during mitosis and arises distinctly in T cell lymphomas originating from an early progenitor stage. Notably, we report amplification of the corresponding syntenic region (11q23) in a subset of human leukemia leading to the overexpression of several cancer genes, including *MLL/KMT2A*. Our findings provide direct evidence that lymphocytes undergo malignant transformation through distinct genome architectural routes that are determined by both RAG-dependent and RAG-independent DNA damage and a block in cell development.

## Highlights

• Lymphomas from RAG2/p53- and p53-deficient mice bear distinct genome architectures
• Block in T cell development leads to 9qA4-5.3 rearrangements and amplifications
• Breakage-fusion-bridge events trigger 9qA4-5.3 aberrations in early T cell lymphomas
• The syntenic region 11q23 is amplified in some human hematological cancers

## Graphical Abstract

**Figure:**
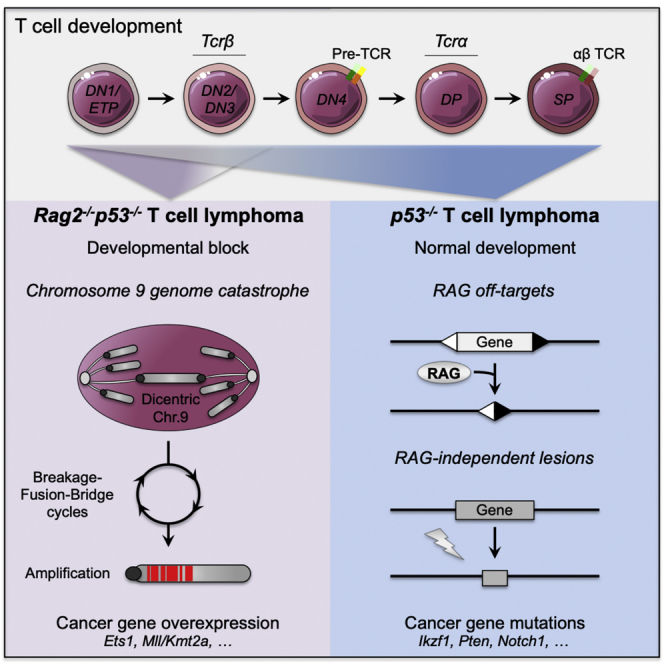

## Teaser

Using p53-deficient T cell lymphoma mouse models, Bianchi et al. show that breakage-fusion-bridge events trigger complex focal genome rearrangements and amplifications in developmentally arrested lymphoblastic cells, leading to unique genome and transcriptome signatures found in some human hematological cancers.

## Introduction

Genetic mutations are thought to result from the combination of specific DNA damage and DNA repair processes that modify the DNA sequence and clonal selection mechanisms that contribute to cancer evolution (Jackson and Bartek, 2009, Stratton et al., 2009, Yates and Campbell, 2012). As DNA damage response and repair genes are quite ubiquitously expressed in all cell and tissue types, it remains puzzling why each cancer subtype harbors a distinctive architecture of genome rearrangements.

Immature lymphoid cells are exposed to potentially harmful DNA damage events as DNA double-strand breaks (DSBs) are generated during the assembly of immunoglobulin (Ig) and T cell receptor (TCR) variable region exons via a cut-and-paste mechanism termed V(D)J (variable [diversity] joining) recombination (Bassing et al., 2002). This process is initiated when the recombination activating gene products RAG1 and RAG2, forming the RAG endonuclease, introduce DSBs between V, D, or J coding gene segments and flanking recombination signal sequences (RSSs). Recombination activating gene (RAG)-induced DSBs activate the ataxia telangiectasia mutated (ATM) kinase-dependent DNA damage response (DDR) that promotes DNA repair and mediates the p53-dependent G1/S checkpoint that arrests or eliminates cells with unrepaired DSBs. Subsequently, the non-homologous end joining (NHEJ) pathway joins RAG-DNA ends in a recombinant configuration, forming a coding joint (the rearranged antigen receptor gene) and a reciprocal signal joint (Deriano and Roth, 2013, Helmink and Sleckman, 2012, Schatz and Swanson, 2011). In addition to promoting adaptive immunity, RAGs have been implicated in the genesis of genetic instability associated with lymphoid malignancy (Marculescu et al., 2006, Onozawa and Aplan, 2012, Roth, 2014).

Loss of p53 alone in mice is sufficient to induce malignancy with a majority of animals developing thymic T cell lymphomas within approximately 4 to 6 months of age (Deriano et al., 2011, Donehower et al., 1992, Jacks et al., 1994, Lescale et al., 2016). In this model, the relative contribution of the RAG recombinase to genomic rearrangements and to overall lymphomagenesis is unclear. Indeed, p53-deficient thymic lymphoma genomes contain a relatively low number of structural variations (SVs), among which only a fraction contains signs of off-target RAG-mediated recombination (Dudgeon et al., 2014, Mijušković et al., 2015). In addition, unlike lymphoid tumors arising in mice expressing mutant RAG proteins (Deriano et al., 2011, Zhang et al., 2011) or harboring DNA repair deficiency (Zha et al., 2010, Zhu et al., 2002), p53-deficient T cell tumors lack recurrent chromosomal translocations or complex genomic rearrangements (Bassing et al., 2003, Deriano et al., 2011, Liao et al., 1998, Mijušković et al., 2015, Morales et al., 2006). Finally, RAGs are not required for lymphomagenesis, as RAG1/p53- or RAG2/p53-doubly deficient mice readily develop thymic T cell lymphomas (Delbridge et al., 2016, Liao et al., 1998, Nacht and Jacks, 1998).

Here, we use deep whole genome sequencing and cytogenetics to analyze the genomes of T cell lymphomas from p53-deficient mice expressing or lacking RAG2. We report that both RAG-dependent and RAG-independent DNA damage underlie the onset of focal DNA deletions in p53-deficient lymphoblastic T cells. Strikingly, in tumors lacking RAG2, we identify a unique genome signature associated with complex genome rearrangements and amplifications of the chromosomal region 9qA4-5.3. We show that this localized genomic catastrophe occurs via a breakage-fusion-bridge mechanism and appears distinctly in T cell lymphomas originating from an early developmental stage. Notably, we also report amplification of this region in a small subset of human acute myeloid leukemia (AML) that is associated with *TP53* alterations and, moreover, amplification of this region in mouse and human tumors leads to the overexpression of several candidate or known cancer genes including *Mll/Kmt2a*.

## Results

### T Cell Lymphomagenesis in *Rag2*^*−/−*^*p53*^*−/−*^ and *p53*^*−/−*^ Mice

**Figure 1:**
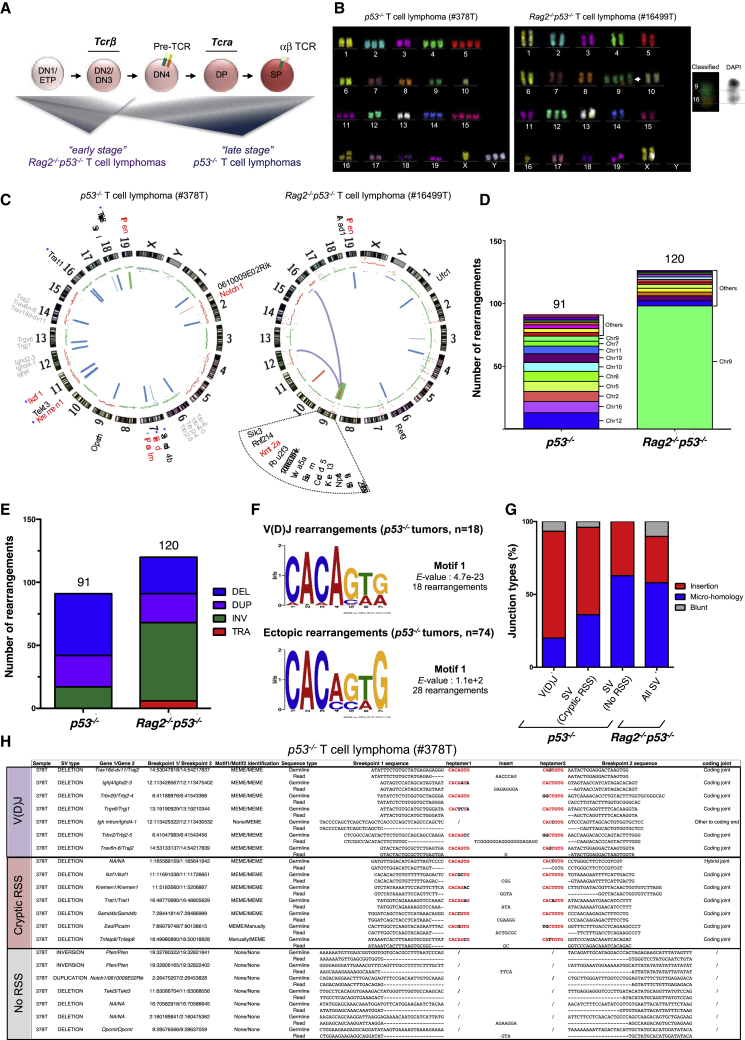
Genome-wide Instability in p53-Deficient T Cell Lymphomas (A) Schematic of T cell development and lymphoma onset; DN, CD4^−^CD8^−^; ETP, early T cell progenitors; DP, CD4^+^CD8^+^; SP, CD4^+^ or CD8^+^. (B) Spectral karyotyping of representative metaphases. White arrow, t(9;16). (C) Circos plots of SVs and CNVs detected in representative tumors. CNV: blue, loss; red, gain. SV: translocation, purple; inversion, green; deletion, blue; duplication, red. Genes altered by SVs are annotated: gray, V(D)J genes; red, cancer genes; blue asterisk, cryptic RSS at the breakpoint; gray asterisk, cryptic RSS at the partner breakpoint. (D) Number and distribution of ectopic SVs in *p53*^*−/−*^ (n = 4) and *Rag2*^*−/−*^*p53*^*−/−*^ (n = 4) lymphomas. (E) Number and type (TRA, translocation; INV, inversion; DUP, duplication; DEL, deletion) of SVs in *p53*^*−/−*^ (n = 4) and *Rag2*^*−/−*^*p53*^*−/−*^ (n = 4) lymphomas. (F) Agnostic motif search of heptamer sequences using Multiple EM for Motif Elicitation (MEME) at the V(D)J and ectopic breakpoint junctions from *p53*^*−/−*^ lymphomas (n = 4). (G) Junction types in canonical V(D)J rearrangements and ectopic SVs from *p53*^*−/−*^ (n = 4) and *Rag2*^*−/−*^*p53*^*−/−*^ (n = 4) tumors. (H) Breakpoint junction sequences from one representative *p53*^*−/−*^ tumor. Germline, mm10 reference genome; read, consensus reads identified at the breakpoint junctions by Delly algorithm. Conserved heptamer nucleotides are in red. For sequences with RSSs, gaps represent resected nucleotides. For sequences without RSSs, only the 20 bp included in the SV are shown for the consensus read. Gaps represent the nucleotides not conserved in the SV. NA, regions not annotated.

To decipher the role of the V(D)J recombinase in lymphomagenesis, we bred *Rag2*^*−/−*^ mice (Shinkai et al., 1992) with *p53*^*−/−*^ mice (Jacks et al., 1994) and followed tumor appearance in *p53*^*−/−*^ and *Rag2*^*−/−*^ *p53*^*−/−*^ mice (Figures S1 and S2). We next analyzed T cell lymphomas originating from these mice at the DNA level by performing whole genome deep sequencing of four *p53*^*−/−*^ and four *Rag2*^*−/−*^*p53*^*−/−*^ thymic T cell tumors at an average coverage of 36X (range = 18−52) (Figure S2; Table S1). As expected for RAG-proficient *p53*^*−/−*^ T cell lymphomas, sequence variation analysis showed alterations in the TCR and Ig genes (range = 2−7 rearrangements per tumor sample), indicating a clonal or oligoclonal origin (Figure S2A; Table S2). The *Tcrβ*, *Tcrα*, and *Igh* gene loci were most frequently rearranged with *Tcrα* rearrangements detected in all four tumor samples (Figure S2A; Table S2), indicating that *p53*^*−/−*^ T cell tumor clone(s) originate from a cluster of differentiation 4 (CD4)^+^/CD8^+^ double positive (DP) and/or single positive (SP) T cell stage (Figure 1A). Consistent with this, p53-deficient T cell tumors harbored high TCRβ expression and high CD28 expression, a marker of T cell differentiation post pre-TCRβ selection (Figure S2B) (Gross et al., 1992). Notably, oncogenic lesions underlying these tumors might originate at an earlier stage, followed by further differentiation to yield the observed phenotype (Figure 1A). In contrast, we did not detect V(D)J rearrangements or CD28/TCRβ expression in *Rag2*^*−/−*^*p53*^*−/−*^ lymphomas owing to the incapacity of developing T cells to initiate V(D)J recombination and differentiate in the absence of RAGs (Figures S2A and S2B; Table S2) (Shinkai et al., 1992). Together with the observation that in young *Rag2*^*−/−*^ *p53*^*−/−*^ animals, T cells are arrested at a CD4^−^/CD8^−^ double negative (DN) stage of differentiation prior to overt cell transformation (Figure S1C) (Nacht and Jacks, 1998), these results indicate that *Rag2*^*−/−*^ *p53*^*−/−*^ T cell cancer clone(s) originate from an early progenitor T cell stage (Figure 1A).

### Genome-wide Instability in p53-Deficient T Cell Lymphomas

We next conducted spectral karyotyping (SKY) on metaphase preparations of early passage tumor T cells (Figure 1B; Table S3). The average chromosome number was 47 (range = 31−66) and 43 (range = 13−70) in *p53*^*−/−*^ and *Rag2*^*−/−*^ *p53*^*−/−*^ individual tumor T cells, respectively (Table S3). Chromosome gains were more frequent than chromosome losses and, although any of the 20 chromosomes could be affected, gains of chromosomes 14 and 15 were found in more than 50% of the metaphases analyzed in both T cell lymphomas (Figure 1B; Table S3), suggesting that one or multiple selection mechanisms shape aneuploidy in p53-deficient tumors. SKY confirmed the rarity of clonal translocations in *p53*^*−/−*^ T cell lymphomas (Deriano et al., 2011), while, more surprisingly, most *Rag2*^*−/−*^ *p53*^*−/−*^ lymphoma analyzed harbored recurrent translocations involving chromosome 9 (Figure 1B; Table S3).

To further characterize genomic instability, we analyzed somatic structural variations (SVs) from our whole genome sequencing data. In total, we identified 211 SVs, including 6 inter-chromosomal and 205 intra-chromosomal rearrangements (Figure S3; Tables S2 and S4). In *p53*^*−/−*^ T cell lymphomas, our analysis identified 91 intra-chromosomal SVs that were quite uniformly dispersed throughout the genome (Figures 1C, 1D, and S4A). These SVs were primarily deletions (49 SVs; 54%), with a relatively equivalent number of duplications (25 SVs; 27%) and inversions (17 SVs; 19%) (Figures 1C, 1E, and S4A; Tables S2 and S4). Although lacking RAG nuclease activity, *Rag2*^*−/−*^ *p53*^*−/−*^ T cell lymphomas harbored a high number of SVs, with 120 rearrangements identified in four tumor samples. Strikingly, in contrast to *p53*^*−/−*^ tumors a vast majority of these rearrangements (98 SVs; 81%) located to chromosome 9, with only 22 SVs distributed throughout the other 19 chromosomes (Figures 1C, 1D, and S4B). In addition, *Rag2*^*−/−*^ *p53*^*−/−*^ T cell lymphoma-associated SVs were primarily inversions (62 SVs; 52%), with the remaining being deletions (29 SVs; 24%), duplications (23 SVs; 19%), and translocations (6 SVs; 5%), which were notably not present in the *p53*^*−/−*^ lymphomas (Figures 1C, 1E, and S4B; Tables S2 and S4). These data demonstrate that the architecture of somatic rearrangements differs greatly between *Rag2*^*−/−*^ *p53*^*−/−*^ and *p53*^*−/−*^ T cell lymphoma genomes.

One candidate driver of genetic instability in RAG-proficient *p53*^*−/−*^ lymphomas is off-target V(D)J recombination at cryptic RSSs (Mijušković et al., 2015). To test this, we computationally resolved 92 rearrangements from *p53*^*−/−*^ tumors to the base-pair resolution and performed an agnostic motif search analysis, looking for a 7-bp motif that corresponds to the size of the well-conserved RSS heptamer in 20 bp of sequence on each side of the corresponding breakpoint junction (Mijušković et al., 2015) (Tables S2 and S4). As expected, analysis of 18 precisely resolved SVs representing canonical V(D)J rearrangements revealed a significant motif (E-value = 4.7 × 10^−23^) corresponding to the perfect RSS heptamer CACAGTG across 32 breakpoint junctions (Figure 1F). In addition, the majority of these rearrangements (73%) contained nucleotide insertions, generated by terminal deoxynucleotidyl transferase (TdT) during processing of RAG-DNA ends (Figure 1G). Analysis of 74 ectopic rearrangements also identified a motif resembling a RSS heptamer sequence CAC(A/C)(C/G)(A/T)(G/C) across 35 breakpoint sequences corresponding to 28 SVs containing a cryptic RSS at one or both ends of the breakpoint junction **(**Figure 1F**)**. Consistent with a RAG origin, 60% of ectopic SVs containing cryptic RSSs had non-templated nucleotide insertions at the breakpoint junctions (Figure 1G). However, unlike canonical V(D)J rearrangements, motif search analysis of ectopic SVs did not reach statistical significance (E-value = 1.1 × 10^+2^) (Figure 1F). In fact, over two-thirds of ectopic SVs (46 SVs) lacked cryptic RSS at breakpoint sequences, and, consistent with a RAG-independent origin, analysis of associated junctions did not reveal a bias toward non-templated nucleotide insertions **(**Figure 1G). To ascertain the reliability of our method, we manually inspected all SVs from tumor sample #378T and confirmed these findings (Figure 1H). Our analysis also revealed ectopic rearrangements affecting multiple known cancer genes, some of which bore the hallmarks of RAG activity (e.g., *Ikzf1*, *Kremen1*), while others lacked RSS-like motifs at breakpoint junctions (e.g., *Notch1*, *Pten*) (Figures 1H and S4A). In addition, although only 22 SVs were identified outside chromosome 9 in *Rag2*^*−/−*^ *p53*^*−/−*^ T cell lymphomas, among these, we identified four rearrangements affecting *Notch1* and *Pten*, confirming that DNA lesions at these cancer genes can occur in the absence of RAG activity (Figure S4B). Altogether, these data demonstrate that both RAG-dependent and RAG-independent mechanisms underlie the formation of oncogenic DNA lesions in *p53*^*−/−*^ T cell lymphomas.

### Chromosome 9 Rearrangements and Amplifications in *Rag2*^*−/−*^*p53*^*−/−*^ T Cell Lymphomas

**Figure 2:**
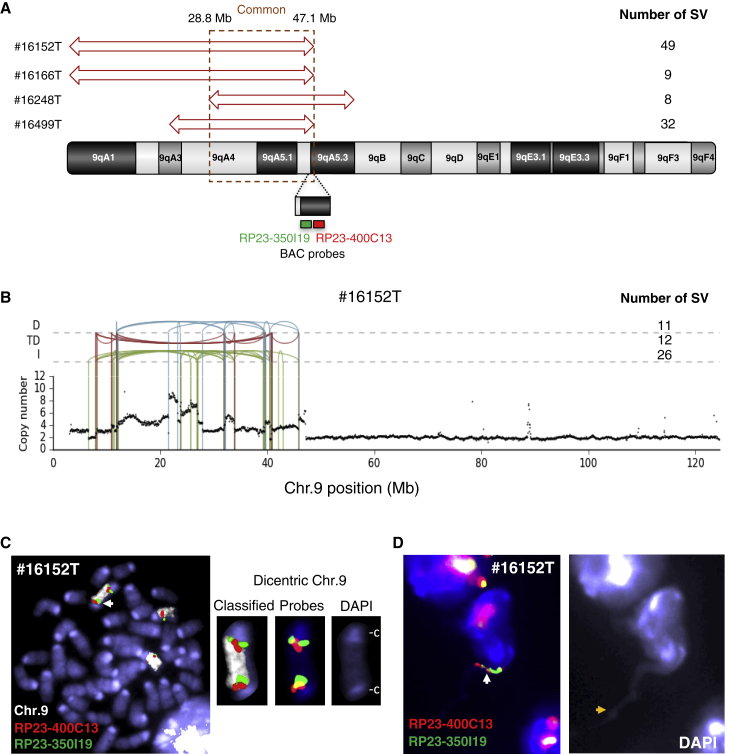
Chromosome 9 Rearrangements and Amplifications in *Rag2*^*−/−*^*p53*^*−/−*^ T Cell Lymphomas (A) Chromosome 9 (chr.9) amplified regions (red arrows) and associated SV numbers in *Rag2*^*−/−*^*p53*^*−/−*^ lymphomas (n = 4). (B) Chr.9 SVs (deletion [D], blue; duplication [TD], red; inversion [I], green) and CNVs from one representative *Rag2*^*−/−*^*p53*^*−/−*^ tumor. (C) Metaphase from one representative *Rag2*^*−/−*^*p53*^*−/−*^ tumor. White arrow, dicentric; c, centromere. (D) Broken chromatin bridge. White arrow, amplified region; yellow arrow, chromatin bridge breakpoint.

Genome-wide analysis revealed chromosome 9 alterations in all four *Rag2*^*−/−*^ *p53*^*−/−*^ T cell lymphomas (Figure S4B; Table S2). The first feature of these alterations was the amplification (range from 3 to 12 copies) of a genomic region of approximately 18 Mb that covers the cytogenetic bands 9qA4 to 9qA5.3 (Figures 2A, 2B, and S5A). The second feature was the tight association between copy number variations (CNVs) and SVs, with 98 SVs localizing to 9qA4-A5.3 amplicons (Figures 2A, 2B, and S5A). These SVs were distinct from *p53*^*−/−*^ lymphoma rearrangements by four apparent criteria: (1) they were primarily intra-chromosomal inversions (59 SVs; 60%) (Figures S5A and S5B); (2) they contained inter-chromosomal rearrangements (5 translocations; 5%) (Figure S5B); (3) they lacked RSS-like motifs at the breakpoint junctions (agnostic motif search, data not shown); and (4) the majority of resolved amplicon-associated SVs showed evidence of short base-pair homology at the breakpoint junction (Figure S5C).

To further analyze the chromosome 9 amplicon, we performed DNA fluorescent *in situ* hybridization (FISH) using bacterial artificial chromosome (BAC) probes locating to the commonly amplified region (Figure 2A) in conjunction with whole-chromosome paint specific for chromosome 9 on eight additional *Rag2*^*−/−*^*p53*^*−/−*^ T cell lymphomas. Cytogenetic analysis confirmed the presence of highly clonal 9qA4-5.3 amplification in six additional tumors (Figure S6A; Table S5).

In total, 10 out of 12 independent *Rag2*^*−/−*^*p53*^*−/−*^ T cell lymphomas (83.3%) carried genomic amplification at the 9qA4-5.3 region (Table S5), indicating that 9qA4-5.3 amplification is a common feature of early T cell *Rag2*^*−/−*^ *p53*^*−/−*^ lymphomas. Cytogenetic studies also revealed the presence of dicentric chromosome 9 with amplifications (Figure 2C). Additionally, we occasionally observed the presence of chromatin strings containing chromosome 9 amplified regions between nearby interphase nuclei, as well as nuclear protrusions that most likely represent fragmented chromosome arms rejoining the nucleus after bridge rupture (Gisselsson et al., 2000, Maciejowski et al., 2015) (Figures 2D and S6B). These unique chromosomal structures are known as breakage-fusion-bridge (BFB) amplification intermediates that are unstable and ultimately give rise to locus-specific complex rearrangements associated with genomic amplification (Gisselsson et al., 2000, Maciejowski et al., 2015, Zha et al., 2010, Zhu et al., 2002). Notably, the prevalence of inversion rearrangements in the 9qA4-5.3 region (Figures S5A and S5B) is consistent with a model in which BFB cycles trigger genomic amplifications at this locus (Bignell et al., 2007, Hillmer et al., 2011, Korbel and Campbell, 2013).

### 9qA4-5.3 Amplification in Early T Cell Lymphomas

**Figure 3:**
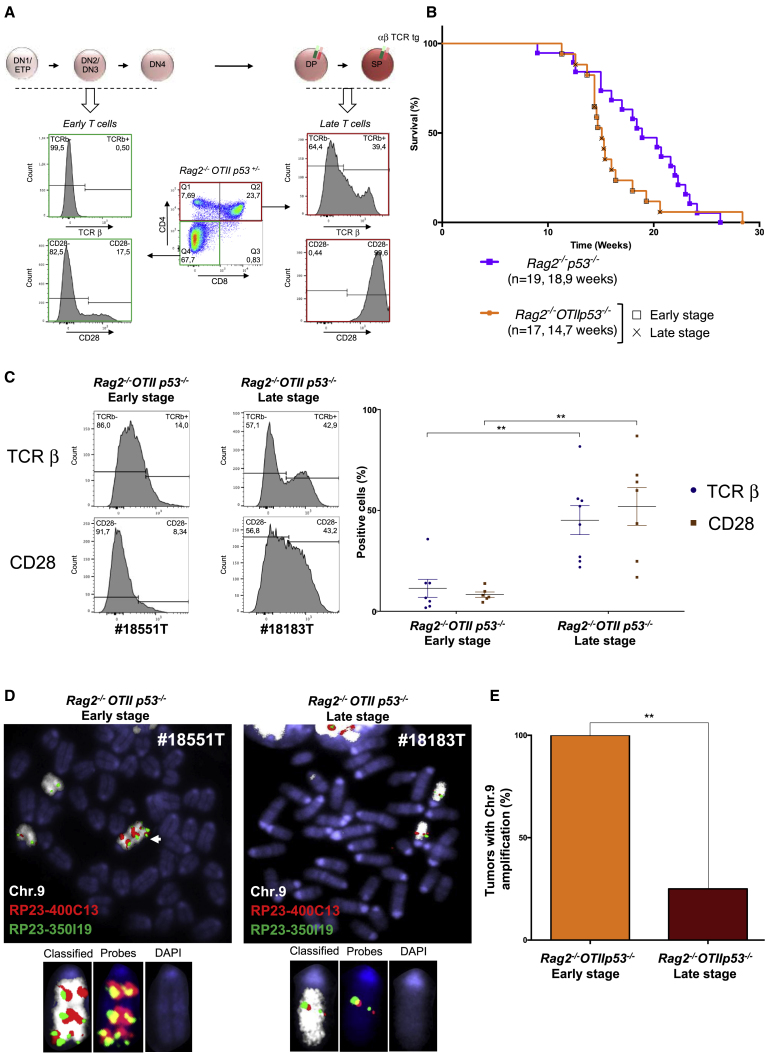
9qA4-5.3 Amplification in Early T Cell Lymphomas (A) TCRβ and CD28 expressions in early (CD4^−^CD8^−^) and late (CD4^+^CD8^+^ and CD4^+^) *Rag2*^*−/−*^*OTII p53*^*+/−*^ T cells. (B) Kaplan−Meier curves of *Rag2*^*−/−*^*p53*^*−/−*^ and *Rag2*^*−/−*^*OTII p53*^*−/−*^ mice. Average age at sacrifice and number of mice analyzed are shown. For *Rag2*^*−/−*^*OTII p53*^*−/−*^ animals: cross, late-stage tumor (n = 7, 15.3 weeks); empty square, early-stage tumor (n = 8, 14.6 weeks). (C) Left, TCRβ and CD28 expression in one early- and one late-stage *Rag2*^*−/−*^*OTII p53*^*−/−*^ T cell lymphoma. Right, percentage of TCRβ and CD28 positive cells in individual *Rag2*^*−/−*^*OTII p53*^*−/−*^ tumors. ^∗∗^p < 0.005, Mann-Whitney test. (D) Metaphases from representative early- and late-stage *Rag2*^*−/−*^*OTII p53*^*−/−*^ lymphomas. White arrow, chr.9 amplification. (E) Percentages of tumors with chr.9 amplification among early-stage (n = 7) and late-stage (n = 8) *Rag2*^*−/−*^*OTII p53*^*−/−*^ T cell lymphomas. ^∗∗^p = 0.007, Fisher exact test.

We reasoned that, in *Rag2*^*−/−*^ *p53*^*−/−*^ mice, developmental block in the context of p53 deficiency might be necessary and sufficient for provoking chromosome 9 instability in precursor p53-deficient T cells lacking RAG activity. To test this hypothesis, we bred *Rag2*^*−/−*^ *p53*^*−/−*^ mice with *Rag2*^*−/−*^ *OTII* transgenic mice (Barnden et al., 1998, Shinkai et al., 1992) to obtain *Rag2*^*−/−*^ *OTII p53*^*−/−*^ mice. The *OTII* transgene leads to the expression of a major histocompatibility complex (MHC) class II-restricted αβ T cell receptor (TCR) under the control of its *Tcrβ* natural regulatory elements, thus enabling T cell differentiation in the absence of V(D)J rearrangements (Barnden et al., 1998). As expected, *Rag2*^*−/−*^ *OTII p53*^*+/−*^ mice completed T cell differentiation with mature CD4^+^/TCRαβ^+^ T cells detected in the thymus (Figure 3A). We monitored a cohort of 17 *Rag2*^*−/−*^ *OTII p53*^*−/−*^ mice for tumor progression. Although not statistically significant, tumorigenesis was accelerated in *Rag2*^*−/−*^ *OTII p53*^*−/−*^ mice (50% survival = 14.7 weeks) relative to *Rag2*^*−/−*^ *p53*^*−/−*^ (50% survival = 18.9 weeks), with all animals developing T cell lymphomas (Figure 3B). Accelerated tumor development in *Rag2*^*−/−*^ *OTII p53*^*−/−*^ animals likely reflects altered thymocyte proliferation in the presence of TCR signaling, as previously reported in RAG1/p53-deficient animals carrying a similar TCRαβ transgene (Liao et al., 1998).

Interestingly, staining of *Rag2*^*−/−*^ *OTII p53*^*−/−*^ lymphoma cells for the transgenic TCRαβ and CD28 and flow cytometry analysis distinguished two types of tumors. Seven tumors expressed low TCRβ and/or CD28 at the cell surface (less than 15% positive cells) (Figures 3C and S7A; Table S5) and thus likely originated from an early T cell developmental stage prior to TCRαβ transgene expression in DP and SP T cells (Figure 3A and (Barnden et al., 1998)). In contrast, eight tumors readily expressed TCRβ and/or CD28 (more than 15% positive cells) (Figures 3C and S7B; Table S5), thus likely originated from a more mature DP or SP stage (Figure 3A; Barnden et al., 1998). We next examined metaphase spreads from these tumors using BAC probes targeting the 9qA4-5.3 amplicon and whole chromosome 9 paint. Strikingly, all seven early-stage *Rag2*^*−/−*^ *OTII p53*^*−/−*^ T cell lymphomas carried the 9qA4-5.3 amplification at very high frequency (range = 80% to 100%, n = 331 metaphases analyzed) (Figures 3D and 3E; Table S5). In sharp contrast, out of eight late-stage *Rag2*^*−/−*^ *OTII p53*^*−/−*^ T cell lymphomas, six lacked clonal 9qA4-5.3 amplification (range = 0% to 7.3%, n = 338 metaphases analyzed) (Figures 3D and 3E; Table S5)**.** Overall, when compared to early-stage *Rag2*^*−/−*^ *OTII p53*^*−/−*^ T cell lymphomas, the frequency of tumors carrying clonal 9qA4-5.3 amplification was significantly lower in late-stage *Rag2*^*−/−*^ *OTII p53*^*−/−*^ T cell lymphomas, as compared to early-stage *Rag2*^*−/−*^ *OTII p53*^*−/−*^ T cell lymphomas (p = 0.007) (Figure 3E). These results show that T cell development block in *Rag2*^*−/−*^ *p53*^*−/−*^ animals contributes to the onset of early T cell lymphomas carrying clonal 9qA4-5.3 amplifications.

### OncoGenomic Analysis of the 9qA4-5.3 Amplicon in Mice and Humans

**Figure 4:**
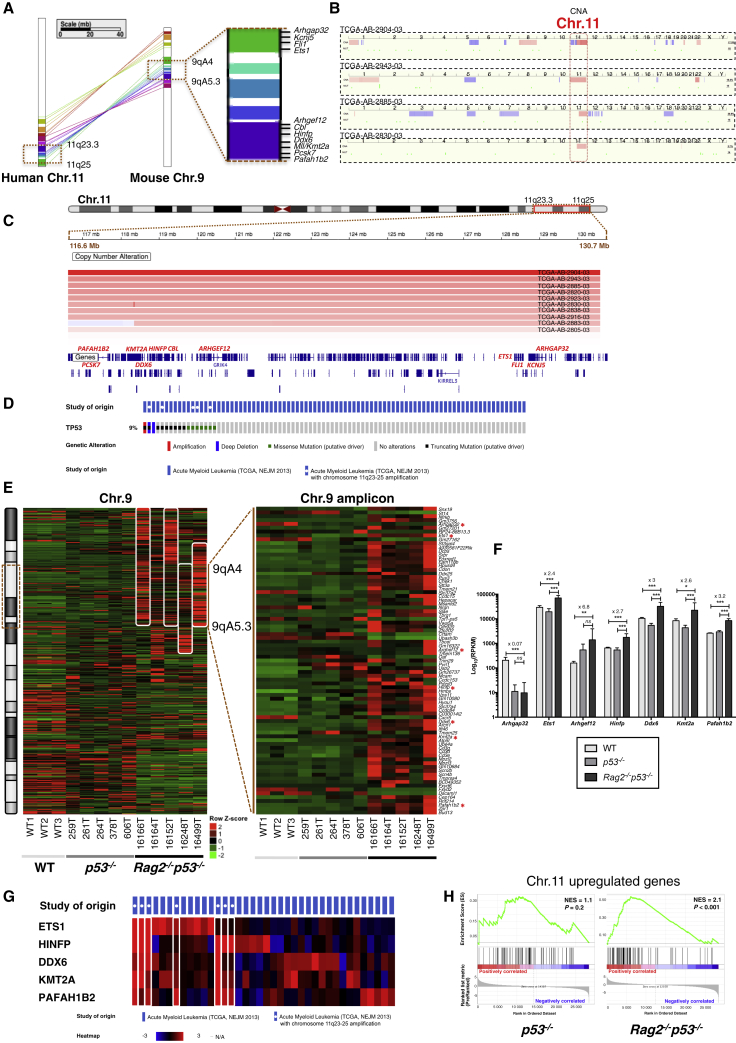
OncoGenomic Analysis of the 9qA4-5.3/11q23-25 Amplicon in Mice and Humans (A) Syntenic map of mouse chr.9 and human chromosome 11 (chr.11) obtained on Cinteny (Sinha and Meller, 2007). Synteny blocks, 11; reversal distance, 2; breakpoint reuse, 1.33. Dashed boxes, mouse chr.9 amplified region transposed to human chr.11. (B) Copy number alteration (CNA) profiles of whole chromosomes for four patients with chr.11 amplification (AML TCGA, The New England Journal of Medicine [NEJM] 2013 – http://www.cbioportal.org). Blue, loss; red, gain. (C) Segmented CNA profiles of the human 11q23.3-25 region from 11 patients (AML TCGA, NEJM 2013). Blue, loss; red, gain. (D) Oncoprint showing mutations and CNAs of *TP53* gene. Not all 170 unaltered patients are shown. (E) Heatmap of RNA sequencing (RNA-seq) (row-normalized) of wild-type (*WT*), *p53*^*−/−*^ and *Rag2*^*−/−*^*p53*^*−/−*^ tumors for the differentially expressed genes in chr.9 and 9qA4-5.3 amplicon. Red asterisk, cancer genes; white rectangle, amplified region. (F) Expression of cancer genes located in the chr.9 amplicon. Expression fold change of *Rag2*^*−/−*^*p53*^*−/−*^ versus WT is shown. p values were adjusted using the Benjamini-Hochberg correction: ^∗^, 0.01 ≤ *padj* < 0.05; ^∗∗^, 0.001 ≤ *padj* < 0.01; ^∗∗∗^, *padj* < 0.001. Error bars indicate the SEM. (G) Heatmap of RNA-seq (row-normalized) for the cancer genes in the 11q23.3-25 region of patients. Only patients with altered mRNA expression (|z score| ≥ 2) for these genes are displayed (38/187 sequenced). (H) Gene set enrichment analysis (GSEA) plots of chr.11 upregulated genes in human AML/MDS with 11q amplification (Zatkova et al., 2009) for *p53*^*−/−*^ and *Rag2*^*−/−*^*p53*^*−/−*^ tumors. NES, normalized enrichment score; p, nominal p value.

Mouse 9qA4-5.3 corresponds to the syntenic 11q23.3-25 region in humans that contains 142 genes, including 11 genes (*ARHGAP32*, *ARHGEF12*, *CBL*, *DDX6*, *ETS1*, *FLI1*, *HINFP*, *KCNJ5*, *MLL/KMT2A*, *PAFAH1B2*, and *PCSK7*) reported within the network of cancer genes database (An et al., 2016) (Figure 4A). 11q23 is frequently rearranged or amplified in hematological malignancies due to the presence of the mixed-lineage leukemia gene (*MLL*, also termed *KMT2A* for lysine [K]-specific methyltransferase 2A), whose alterations represent one of the most common recurring oncogenic events in leukemia (Greaves and Wiemels, 2003, Yunis et al., 1989, Zeisig et al., 2012). While *MLL* aberrations most frequently occur in the form of chromosomal rearrangements leading to the production of chimeric fusions, partial tandem duplications, and internal exonic deletions, studies have revealed amplification of the associated genomic region without characteristic *MLL* rearrangements in approximately 1% of AML and rare cases of myelodysplastic syndrome, as well as acute lymphoblastic leukemia (Greaves and Wiemels, 2003, Yip and So, 2013). Because these studies relied primarily on focus cytogenetics, we thought that the frequency of this amplicon in leukemia might have been underestimated. To test this possibility, we looked for amplification of the region 11q23.3-25 in a series of 187 AML samples for which copy number alteration data were available within The Cancer Genome Atlas (TCGA) database (Ley et al., 2013). We found 11q23.3-25 amplification in 10 samples (5.3%), among which four tumors (2.1%) showed amplification of the whole chromosome, one tumor (0.5%) contained a *MLL*/*MLLT4* fusion associated with amplification of the chromosomal region 3′ of *MLL*, and five tumors (2.7%) displayed partial amplification of chromosome 11 (chr.11) that includes 11q23.3-25 (Figures 4B, 4C, and S8). In analogy to our mouse model, we also looked for the presence of *TP53* mutations in these AML samples. Interestingly, while *TP53* mutations were found in 9% (17/187) of the total AML samples, they were significantly enriched in tumors containing 11q23-25 amplification (5/10, 50%), as compared to tumors lacking 11q23-25 amplification (12/177, 6.8%) (p < 0.001) (Figures 4D and S8). Literature search for leukemia cases reported with 11q23-25 amplifications and for which we could retrieve genetic information on the *TP53* and *MLL* gene status also revealed that *TP53* is frequently altered in 11q23-25 amplified leukemic samples lacking characteristic *MLL* rearrangements (Table S6), as suggested in prior studies (Andersen et al., 2001, Yip and So, 2013, Zatkova et al., 2009). Overall, these data indicate that amplification of the region 11q23-25 is relatively common in human leukemia and occurs preferentially in the context of a defective p53 checkpoint.

Since gene amplification is often found in cancer cells as a mechanism of increasing transcription (Schwab, 1999), we hypothesized that genome amplification in *Rag2*^*−/−*^ *p53*^*−/−*^ tumors might have profound consequences on gene expression. Thus, we performed RNA sequencing on cells from five *Rag2*^*−/−*^ *p53*^*−/−*^ T cell lymphomas, five *p53*^*−/−*^ T cell lymphomas, and three wild-type thymuses. Transcriptome analysis revealed overexpression of multiple 9qA4-5.3 genes in *Rag2*^*−/−*^ *p53*^*−/−*^ tumors (Figure 4E). Among the 11 putative or known cancer genes we identified in the amplicon, seven were differentially expressed in *Rag2*^*−/−*^ *p53*^*−/−*^ tumors (Figure 4E), with five genes overexpressed (*DDX6*, *ETS1*, *HINFP*, *MLL/KMT2A*, and *PAFAH1B2*) in *Rag2*^*−/−*^ *p53*^*−/−*^ tumors as compared to both *p53*^*−/−*^ tumors and wild-type thymocytes (Figure 4F). Interestingly, these five genes were also overexpressed in the TCGA human AML cases with 11q23.3-25 amplification, indicating that amplification of this region leads to similar gene overexpression patterns in mice and humans (Figure 4G). To test this further, we used data from a previous array-based transcriptome analysis of human AMLs that includes cases with or without 11q/*MLL* amplification (Zatkova et al., 2009). In this study, the authors identified 101 genes located on chr.11 that are specifically upregulated in AML cases with 11q/*MLL* amplification. Gene set enrichment analysis revealed that the expression of the majority of these genes was also increased in *Rag2*^*−/−*^ *p53*^*−/−*^ T cell lymphomas but not in *p53*^*−/−*^ T cell lymphomas (Figure 4H), confirming that amplification of the 9qA4-5.3/11q23.3-25 syntenic regions leads to overexpression of multiple common genes in mice and humans.

## Discussion

SVs identified in *p53*^*−/−*^ lymphomas were primarily deletions and carried identifiable features of RAG-induced recombination at breakpoint junctions, with a significant fraction affecting known or candidate cancer genes. Notably, our analysis also revealed multiple rearrangements lacking signs of off-target V(D)J recombination. We envision several explanations for this observation. Our analytical pipeline searches for the presence of cryptic RSSs within a 20-bp window on each side of the breakpoint and is thus unable to detect RSS-like motifs when RAG-generated DNA ends suffer extensive resection prior to joining. In addition, RAG-mediated nicking at bubble-like DNA structures (Raghavan et al., 2004, Tsai et al., 2008) could lead to DNA breakage during replication and, potentially, genomic lesions lacking cryptic RSSs at breakpoint junctions. Alternatively, RAG-independent DNA damage might arise from endogenous cellular processes, which could generate genetic instability. In support of this latter possibility, we occasionally observed the presence of scattered SVs in lymphomas from RAG2/p53-deficient mice. Together, these results demonstrate that both off-target V(D)J recombination and RAG-independent DNA damage underlie the formation of focal DNA lesions in p53-deficient T cell lymphomas.

9qA4-5.3 chromosomal alterations found in *Rag2*^*−/−*^*p53*^*−/−*^ lymphomas are reminiscent of complex rearrangement units, termed amplicons, which were previously reported in the context of combined DNA repair and checkpoint deficiencies, such as in *Atm*^*−/−*^ or *NHEJ*^*−/−*^ *p53*^*−/−*^ mouse lymphomas (Hu et al., 2014, Zha et al., 2010, Zhu et al., 2002) and in some human tumors (Bignell et al., 2007, Campbell et al., 2008, Hillmer et al., 2011, Li et al., 2014, Stephens et al., 2009). Our analysis revealed several features that are compatible with BFB leading to a 9qA4-5.3 amplicon, including the close association of CNVs and SVs within the amplified region, in addition to the vast majority of rearrangements being inversions and breakpoint junctions harboring short base-pair homology, indicative of NHEJ or alternative NHEJ pathways (Bignell et al., 2007, Deriano and Roth, 2013, Hillmer et al., 2011, Korbel and Campbell, 2013). Notably, we observed BFB intermediates containing the 9qA4-5.3 amplicon, such as dicentric chromosomes or chromatin strings, between interphase nuclei (Gisselsson et al., 2000, Maciejowski et al., 2015, Zha et al., 2010, Zhu et al., 2002). These results indicate that in the absence of RAGs, p53-deficient T cells are prone to DNA breakage at 9qA4-5.3, leading to genetic instability and amplification at this locus through BFB.

Using RAG2/p53-deficient animals carrying a TCR αβ transgene that enables rescue of T cell differentiation in the absence of TCR gene rearrangement, we found that 9qA4-5.3 instability is preferentially associated with T cell lymphomas originating from an early developmental stage. These findings are supported by earlier studies reporting 9qA4-5.3 alterations in T cell lymphomas from mice in which inactivation of the TCR β gene enhancer causes a block of T cell development (Haines et al., 2006). In addition, 9qA4-5.3 alterations were occasionally observed in T cell lymphomas arising after transfer of RAG1-deficient thymic cells into a progenitor-deprived “competition-free” recipient thymic environment (Martins et al., 2014). Although it is clear that 9qA4-5.3 is a hotspot for amplification, in all cases, the nature of the initial DNA damaging event leading to 9qA4-5.3 instability remains to be determined. One interesting idea is that developmental stage-specific DNA damage—for instance, due to transcription and/or replication of a specific set of genes (Barlow et al., 2013, Boulianne and Feldhahn, 2018, Boulianne et al., 2017, Schwer et al., 2016, Tubbs and Nussenzweig, 2017)—might predispose 9qA4-5.3 to DNA breakage in early T cells, with subsequent gene amplification selected for during cancerous clonal evolution. As we found 9qA4-5.3 instability in the vast majority of *Rag2*^*−/−*^*p53*^*−/−*^ T cell lymphomas analyzed, *Rag2*^*−/−*^*p53*^*−/−*^ mice provide an elegant model to further investigate the origin of genetic instability in this region.

Mouse 9qA4-5.3 corresponds to the syntenic 11q23.3-25 region in humans that is frequently rearranged or amplified in acute leukemia and that accounts for more than 70% of infant leukemias (Greaves and Wiemels, 2003, Yip and So, 2013, Yunis et al., 1989). This region contains 11 genes, including *MLL*, reported within the network of cancer genes database (An et al., 2016). Interestingly, focal 11q23 amplification without characteristic oncogenic *MLL* fusions (Liu et al., 2009) is thought to occur in approximately 1% of AML and Myelodysplastic syndrome (MDS) and is associated with adverse outcomes (Yip and So, 2013). Through mining the genomes of 187 adult *de novo* AML patients (Ley et al., 2013), we identified 11q23 amplification in 5.3% of the samples, with 2.7% of the cases harboring focal 11q23 amplifications, indicating that genetic instability at this locus might account for a higher number of patients than previously thought. The mechanistic basis for the vulnerability of early hematolymphoid progenitors to these events remains a matter for further investigation.

In addition, whether 11q23 amplification per se is oncogenic is unclear, as there is no *in vivo* disease model for 11q23 amplification. While *MLL* has been suspected to be the main target of 11q23 amplification (Yip and So, 2013), recent analysis of mice overexpressing full-length human *MLL* cDNA indicates the requirement of additional events, possibly genes co-amplified in the 11q23 amplicon, for full-blown leukemia development (Yip et al., 2017). In this regard, we identified five known or candidate cancer genes—*Ddx6*, *Ets1*, *Hinfp*, *Mll*, and *Pafah1b2—*whose expression is significantly upregulated in 9qA4-5.3 amplicon-bearing T cell lymphomas from *Rag2*^*−/−*^ *p53*^*−/−*^ mice. Of note, these genes are also overexpressed in human AML cases harboring the 11q23 amplicon, suggesting that amplification of this region might participate in lymphoid cancer onset and/or progression in both mice and humans.

## STAR★Methods

### Key Resources Table

**Table:** 

REAGENT or RESOURCE	SOURCE	IDENTIFIER
**Antibodies**		

FITC Rat Anti-Mouse CD8b (Ly-3) (Clone YTS156.7.7)	BioLegend	Cat# 126606; RRID:AB_961295
Alexa Fluor 488 Rat Anti-Mouse CD8a (Clone 53-6.7)	BD Biosciences	Cat# 557668; RRID:AB_396780
PE Rat Anti-Mouse CD4 (Clone RM4-5)	BD Biosciences	Cat# 553048; RRID:AB_394584
APC Hamster Anti-Mouse CD3e (Clone 145-2C11)	BD Biosciences	Cat# 553066; RRID:AB_398529
V450 Rat anti-Mouse CD44 (Clone IM7)	BD Biosciences	Cat# 560451; RRID:AB_1645273
PE-Cy7 Rat Anti-Mouse CD25 (Clone PC61)	BD Biosciences	Cat# 552880; RRID:AB_394509
APC eFluor780 Hamster Anti-Mouse TCRb (Clone H57-597)	Thermo Fisher Scientific	Cat# 47-5961-82; RRID:AB_1272173
Alexa Fluor 488 Rat Anti-Mouse CD45R/B220 (clone RA3-6B2)	BD Biosciences	Cat# 557669; RRID:AB_396781
PE Rat Anti-Mouse CD43 (Clone S7)	BD Biosciences	Cat# 553271; RRID:AB_394748
V450 Rat anti-Mouse CD19 (Clone 1D3)	BD Biosciences	Cat# 560375; RRID:AB_1645269
PE-Cy7 Rat anti-Mouse CD117/c-Kit (Clone 2B8)	BD Biosciences	Cat# 558163; RRID:AB_647250
PerCP-Cy5.5 Rat Anti-Mouse IgM (Clone R6-60.2)	BD Biosciences	Cat# 550881; RRID:AB_393944
APC Rat Anti-Mouse IgD (Clone 11-26c.2a)	BD Biosciences	Cat# 560868; RRID:AB_10612002
Rat Anti-Mouse CD16/CD32 (Mouse BD Fc Block) (Clone 2.4G2)	BD Biosciences	Cat# 553142; RRID:AB_394657

**Chemicals, Peptides, and Recombinant Proteins**		

21XMouse - Multicolor FISH Probe for Mouse Chromosomes	MetaSystems Probes	D-0425-060-DI
DAPI/Antifade	MetaSystems Probes	D-0902-500-DA
ProLong Gold Antifade Mountant with DAPI	Thermo Fisher Scientific	P36931
ChromaTide Alexa Fluor 488-5-dUTP	Thermo Fisher Scientific	C11397
ChromaTide Alexa Fluor 594-5-dUTP	Thermo Fisher Scientific	C11400
XMP 9 Orange – Xcyting Mouse Chromosome Paint	MetaSystems Probes	D-1409-050-OR

**Critical Commercial Assays**		

TruSeq DNA PCR-Free High Throughput Library Prep Kit	Illumina	20015963
NEBNext Ultra DNA Library Prep Kit for Illumina	New England Biolabs	E7370
NEXTflex PCR-Free DNA Sequencing Kit	Bioo Scientific	5142-02
TruSeq Stranded mRNA Library Prep	Illumina	20020594
RNeasy Mini Kit	QIAGEN	74104

**Deposited Data**		

Raw data files for RNA sequencing	NCBI Gene Expression Omnibus	GEO:GSE85894
Raw data files for whole genome sequencing	NCBI Sequence Read Archive	SRA: SRP080836

**Experimental Models: Organisms/Strains**		

Mouse: C57BL/6NTac	Taconic	IMSR Cat# TAC:b6; RRID:IMSR_TAC:b6
Mouse: B6.129S2-Trp53^tm1Tyj^/J	The Jackson Laboratory	IMSR Cat# JAX:002101; RRID:IMSR_JAX:002101
Mouse: B6.129S6-Rag2^tm1Fwa^ N12	Taconic	IMSR Cat# TAC:ragn12; RRID:IMSR_TAC:ragn12
Mouse: B6.129S6-Rag2^tm1Fwa^ Tg(TcraTcrb)425Cbn	Taconic	IMSR Cat# TAC:1896; RRID:IMSR_TAC:1896

**Software and Algorithms**		

BWA (v0.7.4)	Li and Durbin, 2009	http://bio-bwa.sourceforge.net/
Picard (v1.94)	Broad Institute	https://broadinstitute.github.io/picard/
GATK (v2.8-1)	DePristo et al., 2011, McKenna et al., 2010	https://software.broadinstitute.org/gatk/
Control-FREEC (v6.3)	Boeva et al., 2012	http://boevalab.com/FREEC/
SVDetect (v0.8b)	Zeitouni et al., 2010	http://svdetect.sourceforge.net/Site/Home.html
Delly (v0.6.7)	Rausch et al., 2012	https://github.com/dellytools/delly
Socrates	Schröder et al., 2014	http://bioinf.wehi.edu.au/socrates/
Meerkat (v0.185)	Yang et al., 2013	http://compbio.med.harvard.edu/Meerkat/
Circos (v0.64)	Krzywinski et al., 2009	http://circos.ca/
MEME (v4.5.0)	Bailey et al., 2006	http://meme-suite.org/
TopHat (v2.0.10)	Kim et al., 2013	https://ccb.jhu.edu/software/tophat/index.shtml
STAR (v2.4.0g1)	Dobin et al., 2013	https://github.com/alexdobin/STAR
HTSeq (v0.6.1)	Anders et al., 2015	https://htseq.readthedocs.io/en/release_0.11.1/
DESeq2 (v1.6.3)	Love et al., 2014	https://bioconductor.org/packages/release/bioc/html/DESeq2.html
SARTools (v1.1.0)	Varet et al., 2016	https://github.com/PF2-pasteur-fr/SARTools
Prism (v6.0)	GraphPad Software	https://www.graphpad.com/scientific-software/prism/
R (v3.0.1)	R Development Core Team	https://www.r-project.org/
GSEA	Broad Institute	http://software.broadinstitute.org/gsea/index.jsp

**Other**		

BAC probe RP23–400C13	Children’s Hospital BACPAC	N/A
BAC probe RP23-350I19	Children’s Hospital BACPAC	N/A
BAC probe RP23-324B12	Children’s Hospital BACPAC	N/A

### Contact for Reagent and Resource Sharing

Further information and requests for reagents may be directed to and will be fulfilled by the Lead Contact, Ludovic Deriano (ludovic.deriano@pasteur.fr).

### Experimental Model Details

We obtained wild-type (Taconic), *p53*^*+/−*^ (Jackson laboratory (Jacks et al., 1994)), *Rag2*^*−/−*^ (Taconic) and *Rag2*^*−/−*^*OTII* (Taconic (Barnden et al., 1998)) mice for this study. *Rag2*^*−/−*^ mice were bred with p53-deficient mice to generate doubly deficient mice. *Rag2*^*−/−*^*OTII* mice were bred with *Rag2*^*−/−*^*/*p53-deficient mice to generate doubly deficient transgenic mice. Male and female animals have been used for this study without noticeable sex bias. Genotyping of mutant mice was performed by PCR of tail DNA as described in the relevant references. All experiments were performed in accordance with the guidelines of the institutional animal care and ethical committee of Institut Pasteur/CETEA n°89 under the protocol numbers 2012-0036 and 180006/14778.

### Method Details

#### Flow cytometry analysis of tumor cells

Lymphoid tumors were analyzed by flow cytometry with antibodies against surface T cell markers (anti-CD4 (RM4–5, 1:200 dilution), anti-CD8 (53-6.7, 1:200 dilution or YTS156.7.7, 1:200 dilution), anti-CD3e (145-2C11, 1:200 dilution), anti-CD44 (IM7, 1:200 dilution), anti-CD25 (PC61, 1:200 dilution), anti-TCRβ (H57-597, 1:200 dilution) and anti-CD28 (E18, 1:200 dilution)) and surface B cell markers (anti-B220 (RA3–6B2, 1:200 dilution), anti-CD43 (S7, 1:150 dilution), anti-CD19 (1D3, 1:200 dilution), anti-IgM (R6–60.2, 1:150 dilution), anti-IgD (11–26c.2a, 1:200 dilution), anti-c-Kit (2B8,1:300 dilution)). Flow cytometry was performed on a FACS Canto II (BD Bioscience) and data were analyzed using FlowJo (TreeStar).

#### Spectral karyotyping of tumor cells

Tumor cells were cultured for 2h in complete RPMI medium before metaphases preparation. Metaphases were prepared as previously described (Lescale et al., 2016). Metaphases spreads were stained with 21X Mouse, Multicolor Painting mFISH Probe Kit (MetaSystems), which was prepared following supplier’s instructions. Slides were mounted in 90% DAPI/Antifade reagent (MetaSystems)/10% ProLong Gold (Thermo Fisher Scientific). Metaphases were imaged using a ZEISS AxioImager.Z2 microscope and the Metafer automated capture system (MetaSystems). Karyotyping was performed using Isis software (MetaSystems).

#### DNA-FISH on metaphase spreads

Metaphases, BAC probes and slides were prepared as previously described (Lescale et al., 2016). BAC probes RP23–400C13 (Chr9:46876752-47065834), RP23-350I19 (Chr9:46653159-46855832) and RP23-324B12 (Chr9:44825064-45042172) were labeled with ChromaTide Alexa Fluor 488- or 594-5-dUTP (Thermo Fisher Scientific as previously described (Chaumeil et al., 2013)). A total of 1 ug of each locus-specific BAC probes were precipitated, pre-annealed, denatured and then mixed with a XCyting Mouse Chromosome 9 (Orange) paint from MetaSystems just before hybridization. Metaphases were imaged using a ZEISS AxioImagerZ.2 microscope and the Metafer automated capture system (MetaSystems), and counted manually.

#### Flow cytometry analysis of T cell development

Lymphocyte development was analyzed in the thymus, bone marrow, lymph nodes and spleen of 4 to 8-week-old mice. All single-cell suspensions were treated with Fc-blocking antibody (CD16–32, 1:200 dilution) before cell surface staining, in phosphate-buffered saline (PBS) with 2% fetal bovine serum for 30 min at 4°C. T lineage cell populations from the thymus were identified based on the expression of the following markers: double-negative (DN) cells (CD4^-^CD8^-^), DN1 (CD4^-^CD8^-^CD44^+^CD25^-^), DN2 (CD4^-^CD8^-^CD44^+^CD25^+^), DN3 (CD4^-^CD8^-^CD44^-^CD25^+^), DN4 (CD4^-^CD8^-^CD44^-^CD25^-^), double-positive (DP) cells (CD4^+^ CD8^+^) and single-positive (SP) cells (CD4^+^CD8^-^ and CD4^-^CD8^+^). T cells from the spleen were identified based on the expression of CD3^+^TCRβ^+^. The following antibodies were used for cell surface staining: anti-CD4 (RM4–5, 1:200 dilution), anti-CD8 (53-6.7, 1:200 dilution or YTS156.7.7, 1:200 dilution), anti-CD3e (145-2C11, 1:200 dilution), anti-CD44 (IM7, 1:200 dilution), anti-CD25 (PC61, 1:200 dilution), anti-TCRβ (H57–597,1:200 dilution) and anti-CD28 (E18, 1:200 dilution). Flow cytometry was performed on a FACS Canto II (BD Bioscience) and data were analyzed using FlowJo (TreeStar).

#### DNA isolation and sequencing

Genomic DNA was prepared from single-cell suspension of mouse lymphomic thymus or healthy kidney using Wizard Genomic DNA purification kit (Promega). Whole-genome DNA libraries were generated with the TruSeq DNA PCR-Free Library Preparation kit (Illumina), NEXTflex PCR-Free DNA-seq kit (Bioo Scientific) or NEBNext Ultra DNA Library Prep (Biolabs). The resulting libraries were then sequenced on an Illumina HiSeq 2000 or HiSeq 2500 using a V3 or V4 flow cell generation generating two 100- or 125- bp paired-end reads. Basecalling was performed with Illumina RTA 1.18.64. Bcl conversions into Fastq were performed using bcl2fastq version 1.8.3. Tumors were sequenced with an average coverage of 36x (range = 18-52) and control samples were sequenced with an average coverage of 15x (range = 11-24).

#### RNA isolation and sequencing

RNA was prepared from single-cell suspensions of mouse lymphomic and healthy thymus using the RNeasy Mini Kit (QIAGEN). Libraries were generated according to the TruSeq Stranded mRNA Library Preparation kit protocol (Illumina). The resulting libraries were then sequenced on an Illumina HiSeq 2000 or HiSeq 2500 using a V3 or V4 flow cell generation generating two 90- or 125- bp paired-end reads. Basecalling was performed with Illumina RTA 1.18.64. Bcl conversions into Fastq were performed using bcl2fastq version 1.8.3. Tumors were sequenced with an average of 100 million paired-end reads per sample.

#### Mapping of Whole Genome sequence reads

Raw sequencing reads were mapped to the reference mouse genome (GRCm38/mm10, Ensembl74) using the Burrows-Wheeler Aligner backtrack algorithm version 0.7.4 (BWA (Li and Durbin, 2009)) with default parameters except the option –q 25 for read trimming. Duplicate reads were removed using the function MarkDuplicates from Picard tools version 1.94 and a filtering for uniquely mapped reads was performed. Reads were subsequently processed with GATK version 2.8-1 (DePristo et al., 2011, McKenna et al., 2010) for indel realignment and base quality score recalibration.

#### Copy number variants analysis

Copy number variants were detected with Control-FREEC algorithm version 6.3, which uses coverage depth differences to identify amplified or deleted regions (Boeva et al., 2012). Read count was calculated in sliding windows (window size was set to 50,000 bp) and control sample was used to normalize read count in the tumor sample. Copy number profiles per chromosome were visualized using R.

#### Identification of SV

Structural Variations (SV) were predicted by SVDetect (Zeitouni et al., 2010) version r0.8b, which uses discordant mapped read pairs provided by the aligner to indicate potential genomic variations from the reference. Mean insert size and standard deviation were computed using the function CollectInsertSizeMetrics from Picard tools. Discordant read pairs with low BWA-backtrack mapping quality scores (the threshold was set to 23) were removed. SVDetect links2compare function was used for comparison of the tumor and control samples, and we disabled the option for comparing only links sharing the same SV type. To control for genetic variation, we subtracted variations found in the genomes of five representative in-house mouse strains (Table S1, mean coverage = 15X, range = 11-24) and 28 other inbred strains of laboratory mice (http://www.sanger.ac.uk/science/data/mouse-genomes-project) (Lescale et al., 2016). We removed SV identified in the genomes of control samples with at least one read pair. We further removed SV not predicted by SVDetect using the BWA-mem discordant read mapping. We removed SV supported with less than 3 read pairs. We used several softwares to identify breakpoints of SV with single-nucleotide resolution: Delly version 0.6.7 (Rausch et al., 2012), Socrates (Schröder et al., 2014) and Meerkat version 0.185 (Yang et al., 2013). We used Socrates and Meerkat to predict micro-homologies and untemplated sequences at breakpoints. We retained intra-chromosomal variants predicted by at least two methods and inter-chromosomal variants predicted by at least three methods. We further selected precisely resolved SV based on one of the following criteria: 1) the SV is annotated « precise » by Delly, 2) the SV is resolved by Socrates, 3) the SV is resolved by Meerkat. SVs were annotated using Ensembl genes GRCm38 release 78. Circos version 0.64 (Krzywinski et al., 2009) plot was used to visualize chromosomal rearrangements and copy number variations. Sanger sequencing confirmed successful PCR amplifications and a breakpoint mapping to base-pair resolution was obtained ((Lescale et al., 2016) and data not shown).

#### Cryptic RSS and V(D)J junction identification

Agnostic repetitive ungapped motif search was performed using MEME version 4.5.0 (Bailey et al., 2006) standard parameters. 20 bp sequences flanking each breakpoint (41 bp spanning the breakpoint junction), of all precisely resolved SV, were screened for heptamer motifs. Only the first most significant motif in each subset is presented. For V(D)J junction identification, one cRSS motif (identified by MEME or manually) should be present in the 41 bp spanning both breakpoints of a rearrangement. The orientation of the heptamer and its position relative to each breakpoint determines the consistency of the rearrangement with RAG cleavage. First, the first three bases of the motif in the CAC orientation should be localized at the right of the first breakpoint involved in the rearrangement, while the first three bases of the motif in the GTG orientation should be on the left of the second breakpoint. Next, to identify the type of junction, we determine whether the heptamer sequences were included or excluded from the junction. Coding joint was characterized by exclusion of both cRSS, while signal joint was identified by inclusion of both cRSS in the junction. For hybrid joint, only one of the two cRSS was included in the junction.

#### Mapping of RNA-seq reads and differential expression analysis

For RNA-seq, mapping was performed with TopHat2 version 2.0.10 (Kim et al., 2013) using Ensembl annotation (GRCm38, release 73) and STAR version 2.4.0g1 using Ensembl annotation (GRCm38, release 78) (Dobin et al., 2013). Duplicate reads were removed using the function MarkDuplicates from Picard tools and a filtering for uniquely mapped reads was performed (for TopHat2, we extracted reads with the tag NH:i:1 and for STAR, we extracted reads with a mapping quality of 255). We used the STAR mapping and quantified read counts for all genes annotated in Ensembl release 78 with HTSeqcount version 0.6.1 (Anders et al., 2015). Differential gene expression analysis was performed with the Bioconductor package DESeq2 version 1.6.3 (Love et al., 2014) and SARTools version 1.1.0 (Varet et al., 2016). p values were calculated by DESeq2 using a Wald test and were corrected for multiple hypothesis testing using the Benjamini-Hochberg correction. Adjusted *P value* of 0.05 was imposed, we used a Log2 Fold-Change cutoff of 1 and a cutoff of minimum 10 reads in *WT* or *Rag2*^*−/−*^*p53*^*−/−*^*.* For heatmap representation, genes were selected from *Rag2*^*−/−*^*p53*^*−/−*^ versus *WT* comparison and their expression levels were converted to row Z-scores. For GSEA enrichment plots, Log2 Fold-Change expression values from *p53*^*−/−*^ and *Rag2*^*−/−*^*p53*^*−/−*^ versus *WT* comparison were used.

### Quantification and Statistical Analysis

Kaplan-Meier mouse tumor-free survival curves were generated and log-rank test was performed for survival and tumor onset analysis using Prism 6.0 (GraphPad Software). Statistical analyses on DNA FISH experiments and for correlation between *TP53* mutations and human 11q23-25 amplification were performed using Fisher exact test with Prism 6.0. For differential expression analysis, *P value*s and corrected *P value*s were calculated with DESeq2 (see above). For all statistical tests, a *P value* of < 0.05 was considered significant (^∗^, 0.01 ≤ p < 0.05; ^∗∗^, 0.001 ≤ p < 0.01; ^∗∗∗^, p < 0.001).

### Data Availability

The whole genome sequencing data have been deposited in NCBI’s Sequence Read Archive under accession number SRP080836 and the transcriptome sequencing data have been deposited in NCBI’s Gene Expression Omnibus under accession number GSE85894.

## Supplemental Information

Document S1. Figures S1−S8 and Tables S1 and S3−S6

Table S2. List of All the Rearrangements Identified in Mouse T Cell Lymphomas, Related to Figures 1 and 2

Document S2. Article plus Supplemental Information
