# Supplementary material for: Breakage-Fusion-Bridge Events Trigger Complex Genome Rearrangements and Amplifications in Developmentally Arrested T Cell Lymphomas
Source: Cell Rep. 2019 Jun 4;27(10):2847–2858.e4. doi: 10.1016/j.celrep.2019.05.014 (PMC6581794; doi:10.1016/j.celrep.2019.05.014)
Supplement: Document S1. Figures S1−S8 and Tables S1 and S3−S6 [file mmc1.pdf]

Cell Reports, Volume *27*

Supplemental Information

Breakage-Fusion-Bridge Events Trigger Complex Genome Rearrangements and Amplifications in Developmentally Arrested T Cell Lymphomas

Joy J. Bianchi, Valentine Murigneux, Marie Bedora-Faure, Chloé Lescale, and Ludovic Deriano

Figure S1

A.

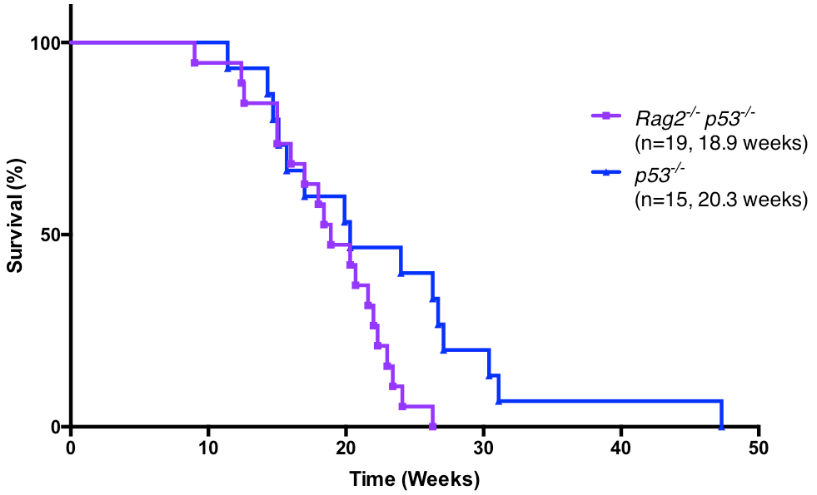

B.

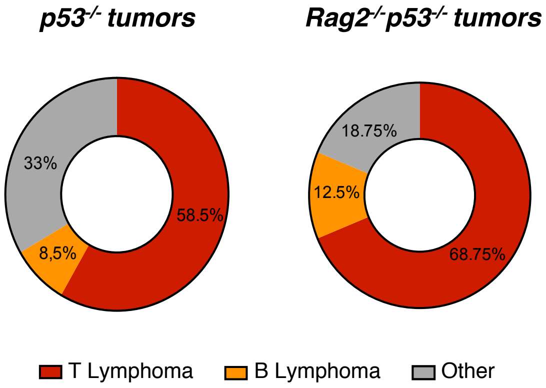

C.

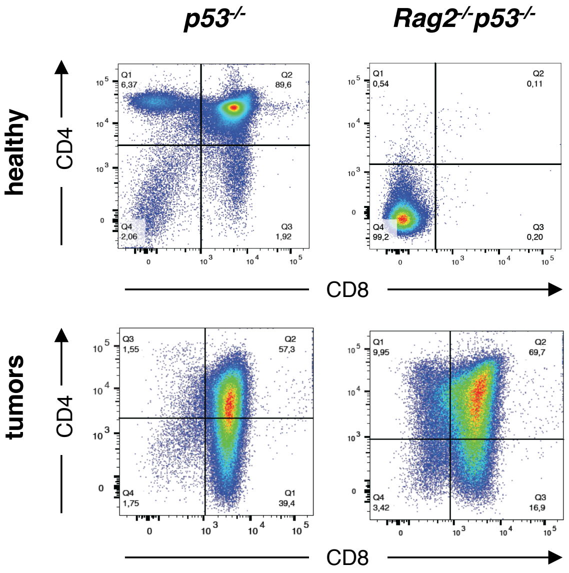

Figure S1. T cell lymphomagenesis in *Rag2$^{-/-}$p53$^{-/-}$* and *p53$^{-/-}$* mice. Related to Figure 1. **A)** Kaplan-Meier survival curves for cohorts of *p53$^{-/-}$* and *Rag2$^{-/-}$p53$^{-/-}$* mice. Animals were monitored for 50 weeks. The average age at sacrifice and number of mice analyzed are shown. **B)** Pie charts showing the tumor spectrum observed for *p53$^{-/-}$* (n=12) and *Rag2$^{-/-}$p53$^{-/-}$* (n=16) animals. « Other » refers to non-lymphoid tumors. **C)** Phenotypic analysis of total thymocytes from one representative healthy (upper) and cancerous thymus (lower) from *p53$^{-/-}$* (left) and *Rag2$^{-/-}$p53$^{-/-}$* (right) animals. CD4 vs CD8 staining profiles are shown.

Figure S2

A.

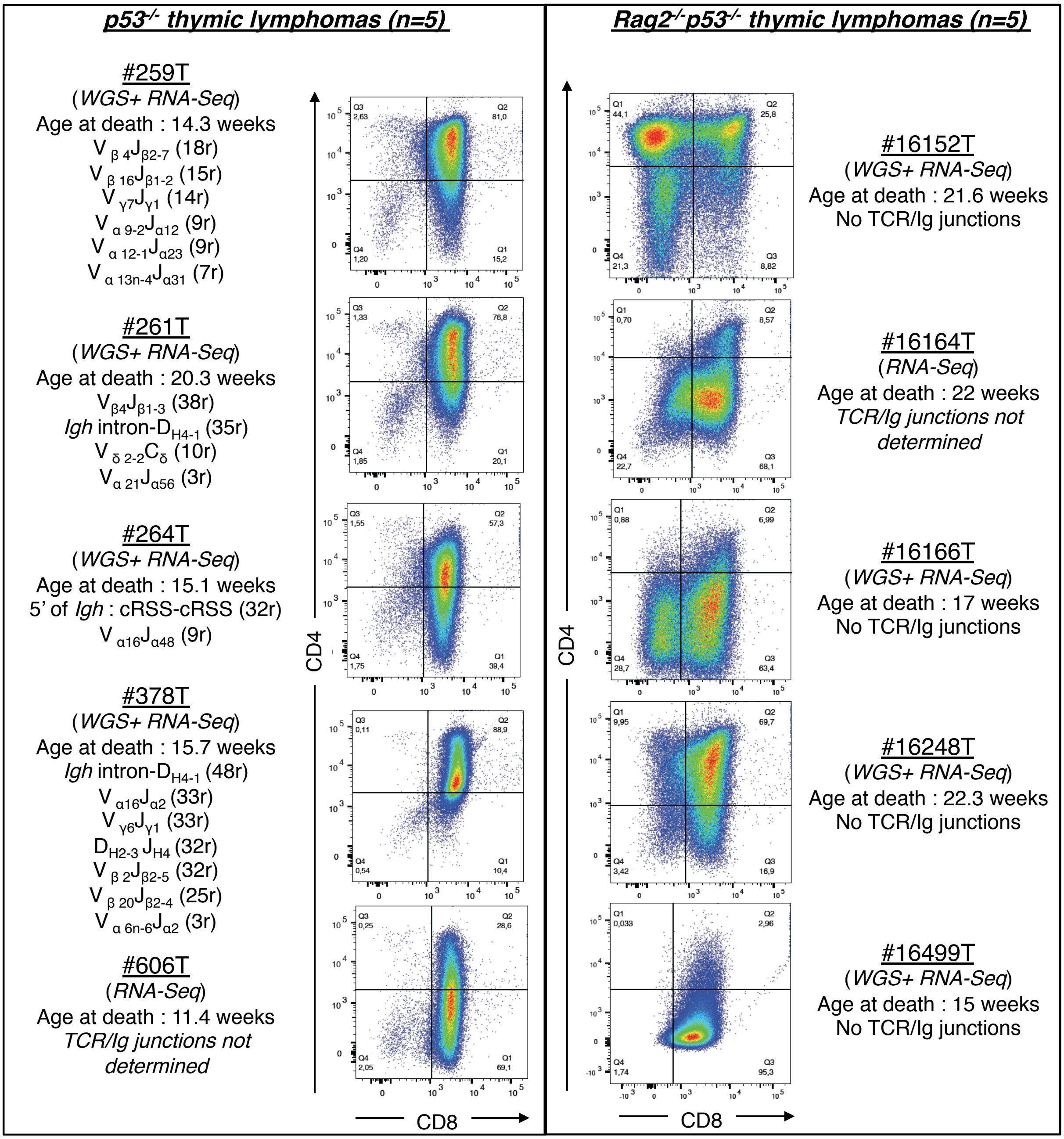

$p53^{-/-}$ thymic lymphomas (n=5)
$Rag2^{-/-}p53^{-/-}$ thymic lymphomas (n=5)
#259T
(WGS+ RNA-Seq)
Age at death : 14.3 weeks
$V_{\beta 4}J_{\beta 2\text{-}7}$ (18r)
$V_{\beta 16}J_{\beta 1\text{-}2}$ (15r)
$V_{\gamma 7}J_{\gamma 1}$ (14r)
$V_{\alpha 9\text{-}2}J_{\alpha 12}$ (9r)
$V_{\alpha 12\text{-}1}J_{\alpha 23}$ (9r)
$V_{\alpha 13n\text{-}4}J_{\alpha 31}$ (7r)
#261T
(WGS+ RNA-Seq)
Age at death : 20.3 weeks
$V_{\beta 4}J_{\beta 1\text{-}3}$ (38r)
Igh intron-$D_{H4\text{-}1}$ (35r)
$V_{\delta 2\text{-}2}C_{\delta}$ (10r)
$V_{\alpha 21}J_{\alpha 56}$ (3r)
#264T
(WGS+ RNA-Seq)
Age at death : 15.1 weeks
5' of Igh : cRSS-cRSS (32r)
$V_{\alpha 16}J_{\alpha 48}$ (9r)
#378T
(WGS+ RNA-Seq)
Age at death : 15.7 weeks
Igh intron-$D_{H4\text{-}1}$ (48r)
$V_{\alpha 16}J_{\alpha 2}$ (33r)
$V_{\gamma 6}J_{\gamma 1}$ (33r)
$D_{H2\text{-}3}J_{H4}$ (32r)
$V_{\beta 2}J_{\beta 2\text{-}5}$ (32r)
$V_{\beta 20}J_{\beta 2\text{-}4}$ (25r)
$V_{\alpha 6n\text{-}6}J_{\alpha 2}$ (3r)
#606T
(RNA-Seq)
Age at death : 11.4 weeks
TCR/Ig junctions not determined
#16152T
(WGS+ RNA-Seq)
Age at death : 21.6 weeks
No TCR/Ig junctions
#16164T
(RNA-Seq)
Age at death : 22 weeks
TCR/Ig junctions not determined
#16166T
(WGS+ RNA-Seq)
Age at death : 17 weeks
No TCR/Ig junctions
#16248T
(WGS+ RNA-Seq)
Age at death : 22.3 weeks
No TCR/Ig junctions
#16499T
(WGS+ RNA-Seq)
Age at death : 15 weeks
No TCR/Ig junctions
CD4
CD8

B.

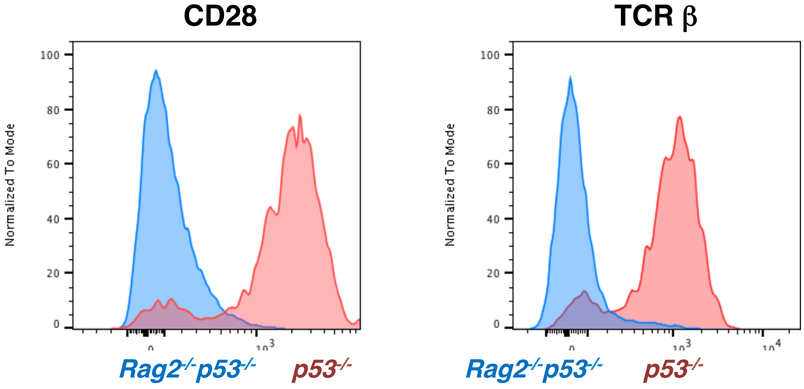

CD28
TCR β
Normalized To Mode
$Rag2^{-/-}p53^{-/-}$
$p53^{-/-}$

Figure S2. Phenotyping and V(D)J rearrangements in sequenced T cell lymphomas. Related to Figure 1. A) CD4 vs CD8 staining profiles of total thymocytes from *p53*$^{-/-}$ and *Rag2*$^{-/-}$*p53*$^{-/-}$ sequenced tumors. Age of mouse at sacrifice and sequencing status (WGS, whole genome sequencing and/or RNA-seq, whole RNA sequencing) are shown on the left. V(D)J rearrangements identified by our structural variants analytic pipeline are listed on the right, followed in brackets by the number of reads supporting each rearrangement. **B)** Staining profiles of CD28 and TCR β of total thymocytes from one representative *p53*$^{-/-}$ tumor (red) and one representative *Rag2*$^{-/-}$ *p53*$^{-/-}$ tumor (blue).

Figure S3

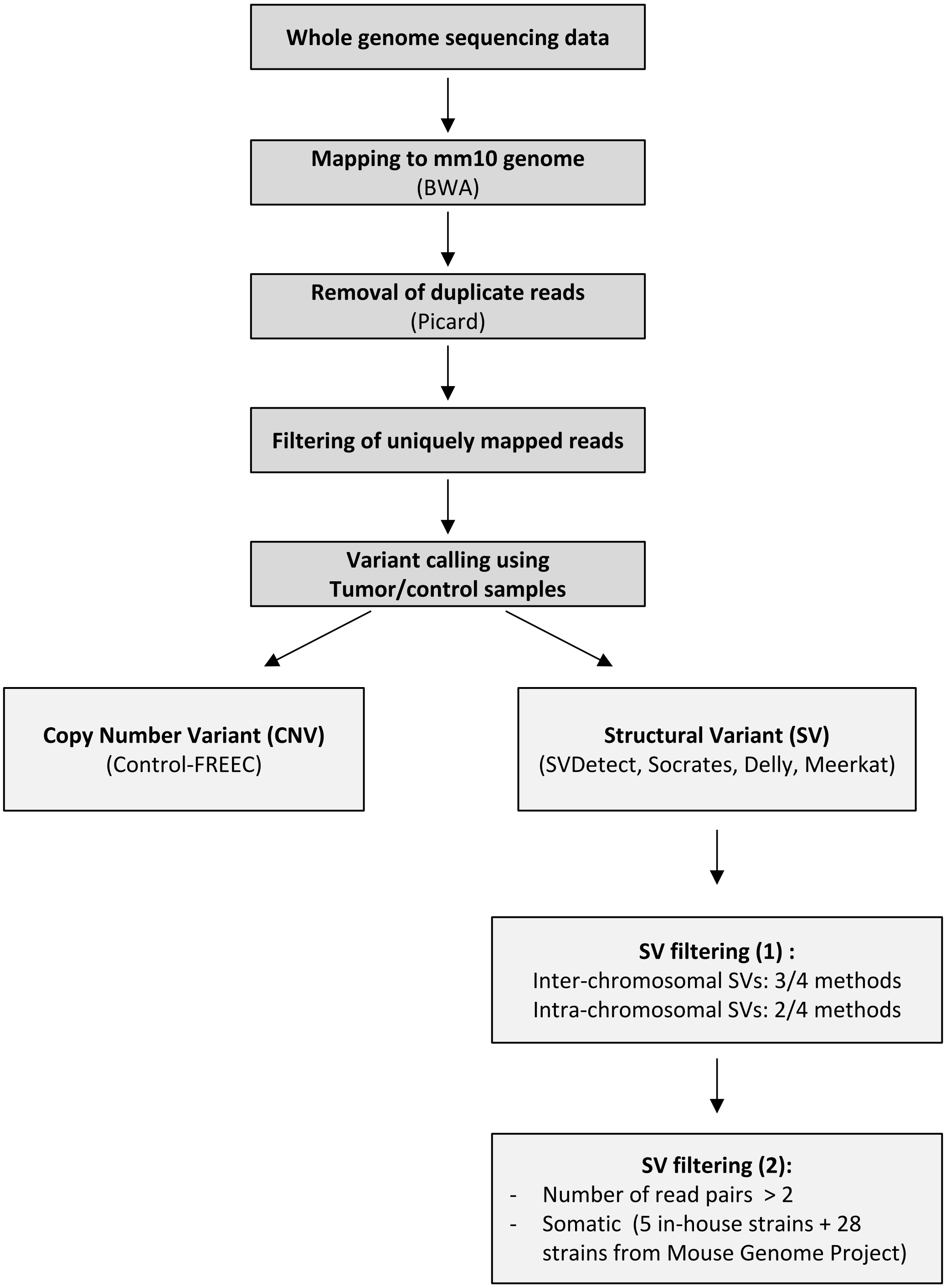

Figure S3. Bioinformatic pipeline to identify copy number and structural variations from whole genome sequencing data. Related to Figures 1 and 2 and STAR Methods. See methods for details.

Figure S4

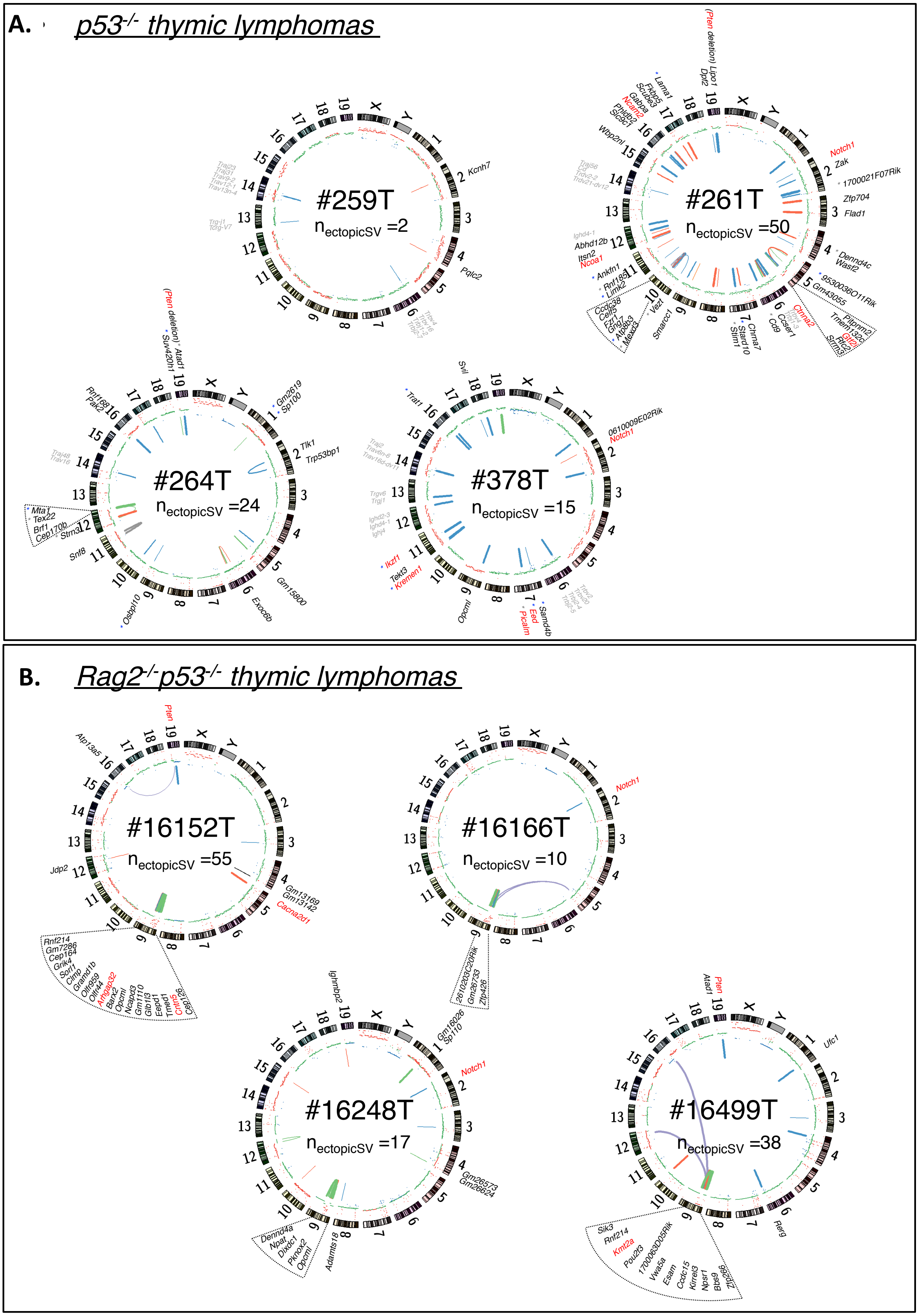
A.
p53-/- thymic lymphomas
#259T
nectopicSV =2
#261T
nectopicSV =50
#264T
nectopicSV =24
#378T
nectopicSV =15
B.
Rag2-/-p53-/- thymic lymphomas
#16152T
nectopicSV =55
#16166T
nectopicSV =10
#16248T
nectopicSV =17
#16499T
nectopicSV =38

Figure S4. Circos plot representation of somatic structural variations. Related to Figure 1. **A)** *p53*$^{-/-}$ (#259T, #261T, #264T, #378T) and **B)** *Rag2*$^{-/-}$*p53*$^{-/-}$ (#16152T, #16166T, #16248T, #16499T) sequenced T cell lymphomas. Chromosomes are arranged circularly end-to-end with each cytoband indicated. Copy number variations are shown along the chromosomes, normalized copy number profile is represented by dots, blue indicating losses and red indicating gains. Structural variations are shown as arcs (translocations in purple, inversions in green, deletions in blue, duplications in red). Genes altered by SV are depicted along the chromosomes. V(D)J segments are represents in gray and known or candidate cancer genes are shown in red. Cryptic RSSs are annotated with blue asterisks when identified at the breakpoint of the indicated gene and with gray asterisks when identified at the partner breakpoint. $n_{ectopicSV}$ indicates the number of ectopic rearrangements locating outside V(D)J sites.

Figure S5

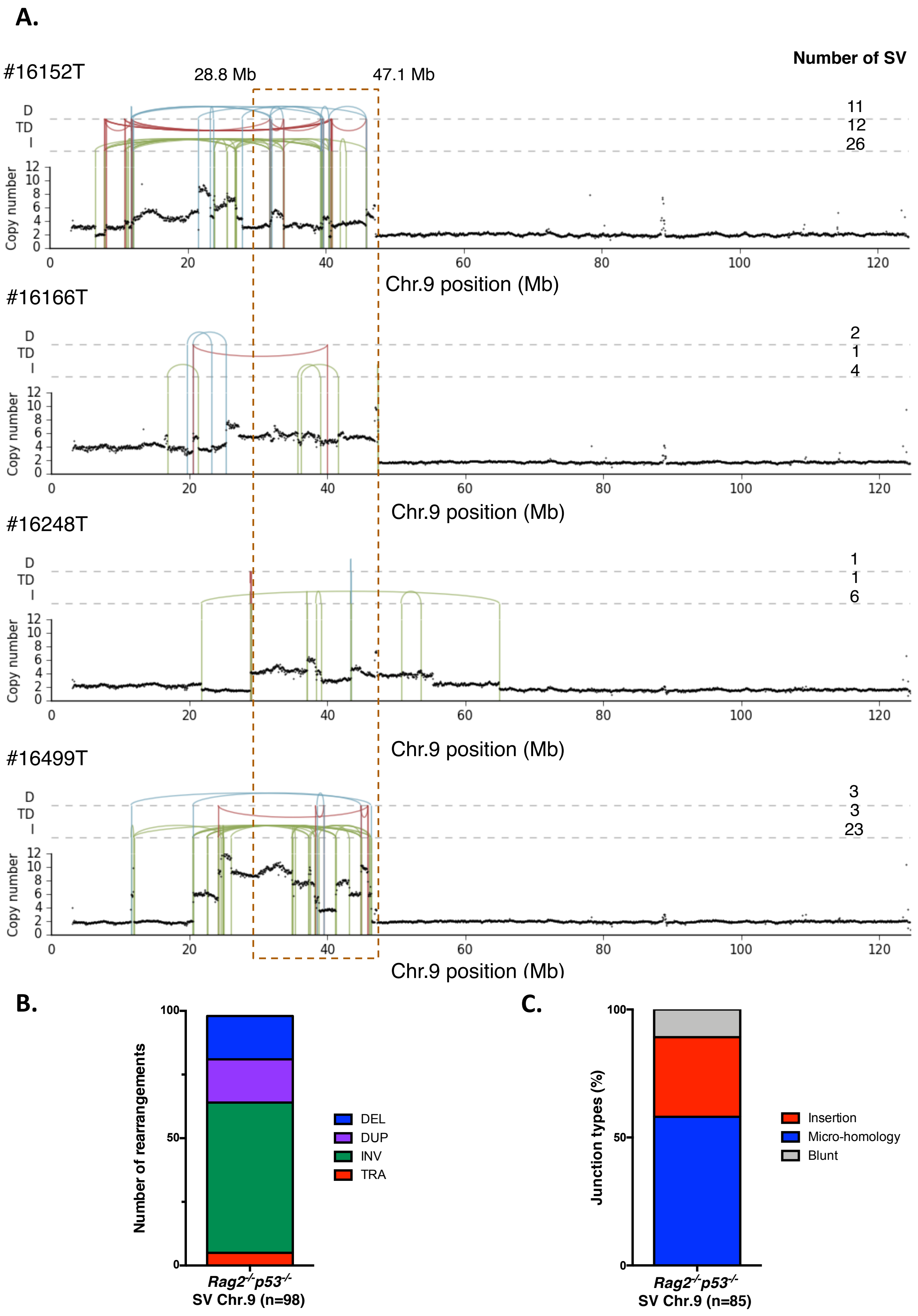
A.
Number of SV
#16152T
28.8 Mb
47.1 Mb
D
TD
I
11
12
26
Copy number
Chr.9 position (Mb)
#16166T
2
1
4
#16248T
1
1
6
#16499T
3
3
23
B.
Number of rearrangements
DEL
DUP
INV
TRA
Rag2-/-p53-/-
SV Chr.9 (n=98)
C.
Junction types (%)
Insertion
Micro-homology
Blunt
Rag2-/-p53-/-
SV Chr.9 (n=85)

Figure S5. Chromosome 9 amplifications and rearrangements. Related to Figure 2. **A)** Rearrangements and copy number variations of chromosome 9 (chr.9) in *Rag2*$^{-/-}$*p53*$^{-/-}$ tumor samples. X-axis represents positions on the chromosome and Y-axis indicates the normalized copy number. Structural variation (SV) types and numbers are indicated: D, deletion (blue); TD, Tandem Duplication (Red); I, Inversion (green). Brown dashed box frames the commonly amplified region. **B)** Bar plots represent the number and type of SVs identified in chr.9 from *Rag2*$^{-/-}$*p53*$^{-/-}$ tumors, (TRA=interchromosomal translocation, INV=inversion, DUP=duplication, DEL=deletion). **C)** Bar plots represent the proportion of SV junction types in chr.9 from *Rag2*$^{-/-}$*p53*$^{-/-}$ tumors.

Figure S6

A.

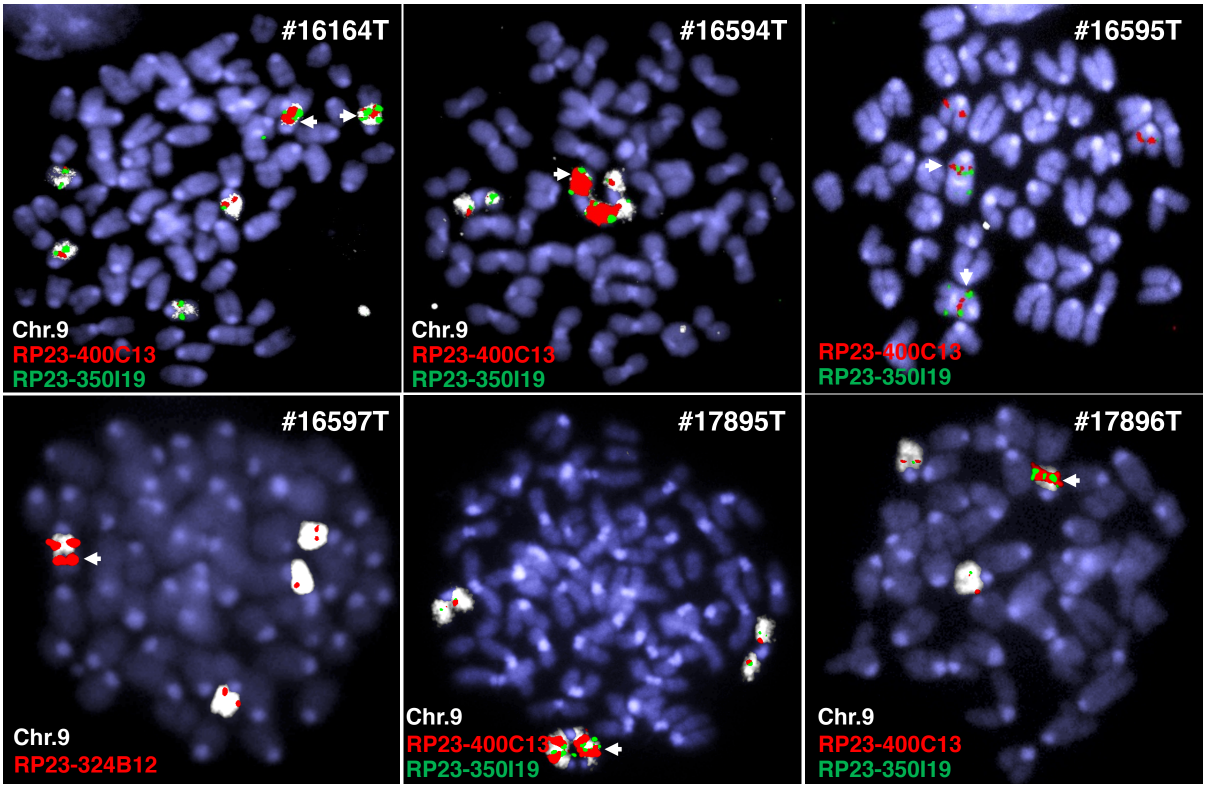
#16164T
Chr.9
RP23-400C13
RP23-350I19
#16594T
Chr.9
RP23-400C13
RP23-350I19
#16595T
RP23-400C13
RP23-350I19
#16597T
Chr.9
RP23-324B12
#17895T
Chr.9
RP23-400C13
RP23-350I19
#17896T
Chr.9
RP23-400C13
RP23-350I19

B.

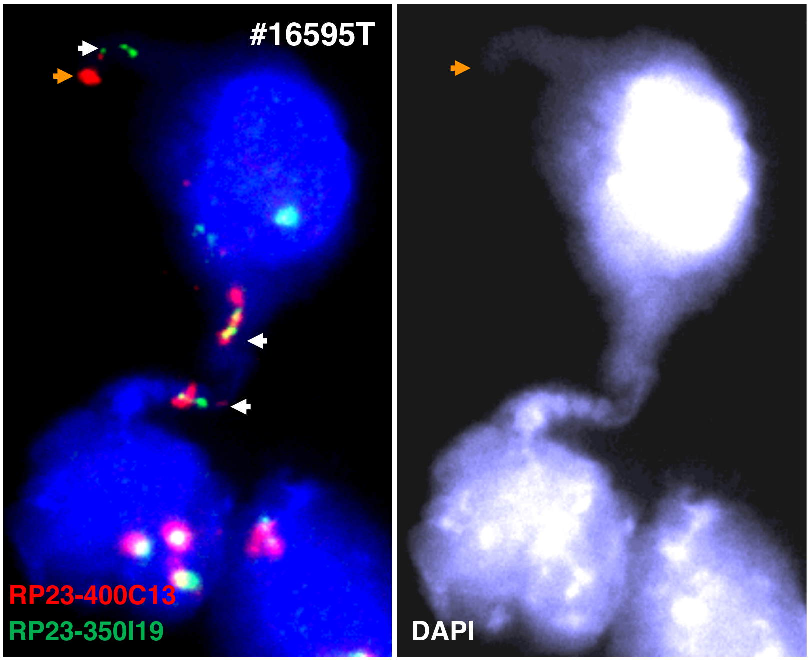
#16595T
RP23-400C13
RP23-350I19
DAPI

Figure S6. Breakage fusion bridge intermediates in *Rag2*$^{-/-}$ *p53*$^{-/-}$ T cell lymphomas. Related to Figure 2. **A)** Representative metaphases from #16164T, #16594T, #16595T, #16597T, #17895T and #17896T *Rag2*$^{-/-}$*p53*$^{-/-}$ T cell lymphomas stained with the BAC probe RP23-400C13 (red), the BAC probe RP23-350I19 (green) or RP23-324B12 (red) and chromosome 9 (chr.9) paint (white). White arrows point at chr.9 amplification. **B)** Representative image of an anaphase bridge and a broken chromatin bridge observed in #16595T *Rag2*$^{-/-}$*p53*$^{-/-}$ tumor. White arrows show the position of chr.9 probes (amplicon) and the yellow arrow points at one broken chromatin bridge.

Figure S7

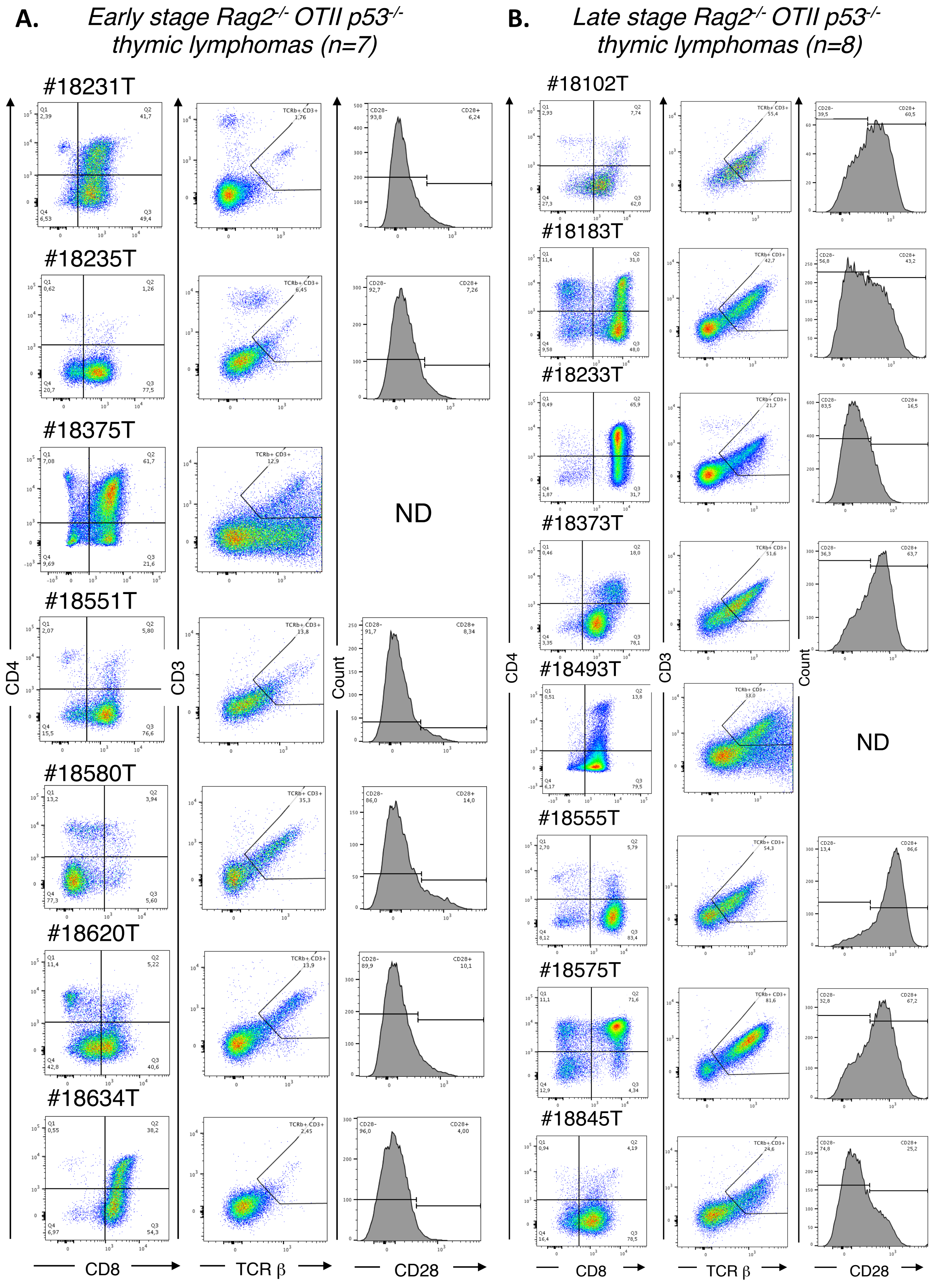
A.
Early stage Rag2-/- OTII p53-/- thymic lymphomas (n=7)
#18231T
#18235T
#18375T
#18551T
#18580T
#18620T
#18634T
ND
CD4
CD3
Count
CD8
TCR β
CD28
B.
Late stage Rag2-/- OTII p53-/- thymic lymphomas (n=8)
#18102T
#18183T
#18233T
#18373T
#18493T
#18555T
#18575T
#18845T
ND
CD4
CD3
Count
CD8
TCR β
CD28

Figure S7. Tumor phenotypes of TCR transgenic mice. Related to Figure 3. CD4 vs CD8, CD3 vs TCRβ and CD28 staining profiles of total thymocytes from **A)** early and **B)** late stage *Rag2*$^{-/-}$ *OTII p53*$^{-/-}$ tumors. ND, not determined. n indicates the number of tumors analyzed.

Figure S8

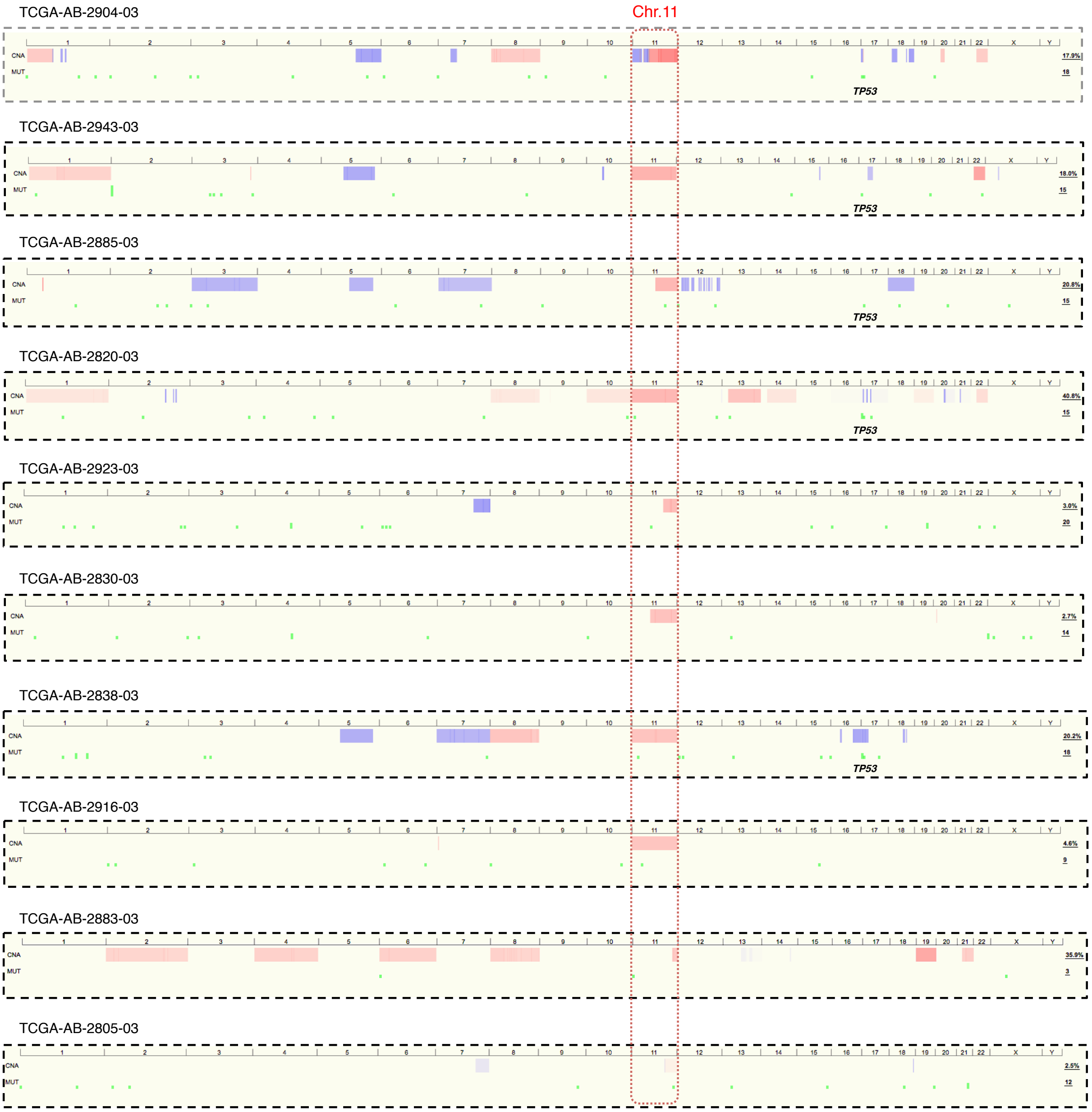

Figure S8. **Chromosome 11 amplification in AML patients.** Related to Figure 4. Copy Number Alteration (CNA) profiles of whole chromosomes for eleven patients presenting amplification of chr.11, in one AML TCGA, NEJM 2013 study extracted from cBioPortal (http://www.cbioportal.org). Blue indicates losses and red indicates gains. Red dashed boxe frames human chr.11. Patients with *TP53* mutations are indicated.

Table S1

Sample ID	Genotype	Raw reads	Mapped reads	Mapped reads (%)	Duplicate reads (%)	Mapped reads (after duplicate removal)	Sequencing coverage (x)
259T	*p53-/-*	6,13E+08	563587666	91,9	8,5	515844208	19,1
261T	*p53-/-*	1,61E+09	1556661704	96,9	10,8	1388462192	51,4
264T	*p53-/-*	1,23E+09	1176770735	95,4	11,5	1041430474	38,6
378T	*p53-/-*	1,61E+09	1565860475	97,2	10,2	1405673989	52,1
16152T	*Rag2-/- p53-/-*	1,17E+09	1119461302	95,5	10,2	1005420525	37,2
16166T	*Rag2-/- p53-/-*	5,7E+08	538525169	94,4	8,3	493795685	18,3
16248T	*Rag2-/- p53-/-*	1,08E+09	1027522497	95,3	11,6	908153233	33,6
16499T	*Rag2-/- p53-/-*	1,23E+09	1171794147	95,3	11,7	1034565694	38,3
14769K	WT	4,38E+08	423694396	96,7	9,9	381848747	14,1
14783K	*Rag2c/c*	4,22E+08	407510759	96,6	9,9	367320198	13,6
14488K	WT	3,95E+08	380529549	96,4	9,9	342726292	12,7
15510K	*Rag2c/c Cernu-/- p53+/-*	7,49E+08	723272106	96,6	9,4	655405964	24,3
17002K	*Rag2-/- p53+/-*	3,64E+08	331706511	91,1	9,9	299029292	11,1

Table S1. Sequencing read counts and statistics. Related to Figures 1, 2 and 4. Raw sequencing reads were mapped to the reference mouse genome using BWA and duplicate reads were removed with the function MarkDuplicates from Picard tools (see Methods).

Table S3

	SKY-MultiFISH		Aneuploidy					
Genotype	Samples	Translocations (Frequency)*	Total number of metaphase examined	Range (average)	Aneuploid events	Aneuploidy/metaphase	Aneuploid chromosomes	Frequency
p53-/-	259T	t(16;14) (2/9)	83	31-66 (47)	662	7,98	1	33/83
							2	40/83
							3	10/83
							4	**54/83**
	261T	(0/21)					5	**72/83**
							6	20/83
							7	10/83
							8	10/83
	264T	t(8;16) (16/21)					9	**42/83**
							10	20/83
							11	**62/83**
							12	**49/83**
	378T	(0/32)					13	14/83
							14	**57/83**
							15	**44/83**
							16	23/83
	606T	ND					17	10/83
							18	12/83
							19	14/83
							X	23/83
							Y	**43/83**
Rag2-/-p53-/-	16152T	t(19;16) (7/20)	97	13-70 (43)	1018	10,49	1	**58/97**
		t(12;11) (6/20)					2	37/97
		t(9;16) (4/20)					3	46/97
							4	**54/97**
	16164T	t(9;15) (16/21)					5	39/97
							6	46/97
							7	45/97
							8	39/97
	16166T	(0/21)					9	**66/97**
							10	**60/97**
							11	**49/97**
							12	**61/97**
	16248T	t(9;4) (12/21)					13	36/97
							14	**71/97**
							15	**80/97**
							16	**64/97**
	16499T	t(9;16) (13/14)					17	35/97
							18	39/97
							19	**56/97**
							X	38/97
							Y	29/97

Table S3. Translocations and aneuploidy in T cell lymphomas. Related to Figure 1. SKY-MultiFISH = Spectral Karyotyping-multi fluorescent in-situ hybridization analysis has been performed to identify gross rearrangements at the cytogenetic level. *Only recurrent translocations (>20%) are shown. t, translocation (the chromosome donating the centromere is listed first). Aneuploidy frequencies (gains and losses) > 50% are shown in red bold.

Table S4

				p53-/-					*Rag2-/-p53-/-*					
				259T	261T	264T	378T	Total *p53-/-*	16152T	16166T	16248T	16499T	Total *Rag2-/-p53-/-*	Total *p53-/-* & *Rag2-/-p53-/-*
All	Intra	V(D)J		6	4	2	7	19	0	0	0	0	0	19
		Ectopic	DEL	0	25	12	12	49	14	3	4	8	29	78
			DUP	2	19	3	1	25	14	1	4	4	23	48
			INV	0	6	9	2	17	26	4	9	23	62	79
	Inter	Translocation		0	0	0	0	0	1	2	0	3	6	6
	Total Intra & Inter			8	54	26	22	110	55	10	17	38	120	230
	Total Intra & Inter ectopic			2	50	24	15	91	55	10	17	38	120	211
Precisely resolved	Intra	V(D)J		6	3	2	7	18	0	0	0	0	0	18
		Ectopic	DEL	0	20	11	11	42	10	3	4	7	24	66
			DUP	0	17	2	1	20	10	1	4	4	19	39
			INV	0	1	9	2	12	22	4	7	22	55	67
	Inter	Translocation		0	0	0	0	0	1	2	0	2	5	5
	Total Intra & Inter			6	41	24	21	92	43	10	15	35	103	195
	Total Intra & Inter ectopic			0	38	22	14	74	43	10	15	35	103	177

Table S4. Status and type of rearrangements identified in T cell lymphomas. Related to Figure 1. The number of SVs is shown for each sample according to their status (intrachromosomal: V(D)J or ectopic rearrangements and interchromosomal rearrangements) and their type (DEL=deletion, DUP=duplication, INV=inversion and translocation, based on Delly annotation). Precisely resolved SVs have their breakpoint bioinformatically resolved at the base-pair resolution (see Methods). For a detailed list of all the SVs, see Table S2.

Table S5

Sample	Genotype	Mouse age at death (weeks)	TCR-b/CD3 expression	CD28 expression	Metaphases examined	Amplification Frequency
16152T	*Rag2-/-p53-/-*	21.6	0.1%	ND	67 (WGS)	53/67* (Yes WGS)
16164T	*Rag2-/-p53-/-*	22	1.1%	ND	50	39/50*
16166T	*Rag2-/-p53-/-*	17	0.2%	ND	ND (WGS)	ND (Yes WGS)
16248T	*Rag2-/-p53-/-*	22.3	0.1%	ND	ND (WGS)	ND (Yes WGS)
16499T	*Rag2-/-p53-/-*	15	0.1%	ND	ND (WGS)	ND (Yes WGS)
16594T	*Rag2-/-p53-/-*	18	0.1%	ND	60	58/60*
16595T	*Rag2-/-p53-/-*	18.9	0%	ND	59	56/59*
16597T	*Rag2-/-p53-/-*	12.4	0.1%	ND	46	43/46*
16446T	*Rag2-/-p53-/-*	20.3	0.1%	ND	66	0/66
16592T	*Rag2-/-p53-/-*	20.7	0%	ND	67	1/67
17895T	*Rag2-/-p53-/-*	15.7	0.1%	ND	50	31/50*
17896T	*Rag2-/-p53-/-*	15	0.6%	ND	32	31/32*
18231T	*Rag2-/-OT2p53-/-*	15.3	2%	6%	77	76/77*
18235T	*Rag2-/-OT2p53-/-*	16	7%	7%	52	49/52*
18375T	*Rag2-/-OT2p53-/-*	20.6	5%	ND	53	48/53*
18551T	*Rag2-/-OT2p53-/-*	15.1	14%	8%	29	29/29*
18580T**	*Rag2-/-OT2p53-/-*	13.7	36%	14%	54	51/54*
18620T	*Rag2-/-OT2p53-/-*	15.4	14%	10%	10	8/10*
18634T	*Rag2-/-OT2p53-/-*	12.6	3%	4%	56	53/56*
18102T	*Rag2-/-OT2p53-/-*	14.4	56%	60%	52	52/60*
18183T	*Rag2-/-OT2p53-/-*	14.4	43%	43%	71	0/71
18233T	*Rag2-/-OT2p53-/-*	19.3	22%	17%	9	0/9
18373T	*Rag2-/-OT2p53-/-*	18	52%	64%	55	4/55
18493T	*Rag2-/-OT2p53-/-*	11.3	27%	ND	70	0/70
18555T	*Rag2-/-OT2p53-/-*	14.3	55%	87%	49	0/49
18575T	*Rag2-/-OT2p53-/-*	14.7	82%	67%	55	52/55*
18845T	*Rag2-/-OT2p53-/-*	16.4	25%	25%	84	0/84

Table S5. Chromosome 9 instability in RAG-deficient T cell lymphomas. Related to Figures 2 and 3. Lymphomas were analyzed by FISH using the BAC probe RP23-400C13 (Chr9:46876752-47065834) combined with BAC probe RP23-350I19 (Chr9:46653159-46855832), except for #16597T for which amplification was observed using the BAC probe RP23-324B12 (Chr9:44825064-45042172). Number of metaphases examined and frequencies of chr.9 amplification are shown. Tumors are considered positive for TCR-β/CD3 or CD28 expression when more than 15% of the cells expressed the cell surface markers. *Tumors harboring recurrent chromosome 9 amplifications (amplifications found in > 20% of the metaphases analyzed).** Tumor #18580T has TCR-β+/CD3+ expression > 15%, however based on its CD28 expression <15% and its $CD4^-/CD8^-$ profile (see Fig.S7), it was classified as an early stage tumor. ND, not determined. Yes WGS, chromosome 9 amplifications were identified by Copy Number Variant analysis after whole genome sequencing (WGS).

Table S6

References	Cancer type	11q23-25 amplified cases<br>Number of cases	*MLL* status<br>Amplification (Frequency)	Rearrangement	*TP53* status*	Methods of analysis
Allen, R.J. et al., Leukemia, 1998	AML	1/1	Yes (1/1)	Not Detected	Loss of chromosome 17 (1/1)	Karyotyping/FISH/Southern Blot/Northern Blot/Western Blot
Amakawa, R. et al., J Clin Exp Hematop, 2010	Acute MLL	1/1	Yes (1/1)	Not Detected	Mutated (1/1)	Karyotyping/FISH/Southern Blot/PCR-SSCP
Andersen, M.K. et al., Genes Chromosomes Cancer, 2001	AML/MDS	12/70	Yes (12/12)	Not Detected	Mutated (7/8)	Karyotyping/FISH/Southern Blot/CGH
Arnaud, B. et al., Cancer Genet Cytogenet, 2005	AML	20/239 (trisomy 11 (7/20), tetrasomy 11 (1/20))	Yes (20/20)	Not Detected	Loss of chromosome 17 (7/20) (11 trisomy (2/7))	Karyotyping/FISH
Avet-Loiseau, H. et al., Genes Chromosomes Cancer, 1999	AML	3/181	Yes (3/3)	Not Detected	Loss of chromosome 17 (1/3)	Karyotyping/FISH
Felix, C.A. et al., Blood, 1998	AML	1/1	Yes (1/1)	Not Detected	Mutated (1/1)	Karyotyping/FISH/Southern Blot/PCR-SSCP
Bajaj, R. et al., Mol Cytogenet, 2011	AML/MDS	2/30	Yes (2/2)	Not Detected	Mutated (1/2)	Karyotyping/FISH/CGH
Cox, C. et al., British Journal of Haematology, 2003	AML	1/289	Yes (1/1)	Not Detected	Loss of chromosome 17 (1/1)	Karyotyping/FISH
Espinet, B. et al., Haematologica, 2003	pre-B ALL	1/1	Yes (1/1)	Not Detected	Loss of chromosome 17 (1/1)	Karyotyping/FISH
Fernandes, C.M. et al., BJH, 2015	AML	1/1	Yes (1/1)	Not Detected	Mutated (1/1)	Karyotyping/FISH
Huh, Y.O. et al., Cancer Genetics, 2016	AML/MDS/CML	4/18	Yes (4/4)	Not Detected	Mutated (3/4)	Karyotyping/FISH
Kim, M.H. et al., Cancer Genet Cytogenet, 2001	AML/MDS	1/19	Yes (1/1)	Not Detected	Loss of chromsome 17p (1/1)	Karyotyping/FISH/CGH
Koka, R. et al., Leukemia & Lymphoma, 2016	AML	1/1	Yes (1/1)	Not Detected	Mutated (1/1)	Karyotyping/FISH/SNP-array/Sequencing
Maitta, R.W. et al., Cancer Genet Cytogenet, 2009	AML	1/1	Yes (1/1)	Not Detected	Loss of chromosome 17 (1/1)	Karyotyping/FISH
Mrozek, K. et al.,Genes Chromosomes Cancer, 2002	AML	7/29 (partial 11q trisomy (3/7))	Yes (7/7)	Not Detected	Loss of chromosome 17 (2/7) (11q partial trisomy (0/2))	Karyotyping/FISH
Papenhausen, P.R. et al., Exp Mol Pathol, 2005	AML/MDS	29/29	Yes (29/29)	Not Detected	Loss of chromosome 17 or 17p (15/29)	Karyotyping/FISH
Park, J.P. et al., Cancer Genet Cytogenet, 2000	AML	2/2	Yes (2/2)	Not Detected	Loss of chromosome 17p (1/2)	Karyotyping/FISH/Southern Blot
Poppe, B. et al., Blood, 2004	AML/MDS	31/31	Yes (31/31)	Not Detected	Loss of chromosome 17 (9/31)	Karyotyping/FISH/qPCR
Rayeroux, K.C. et al., Cancer Gene Cytogenet, 2009	AML/MDS	10/27	Yes (8/10)	Not Detected	Loss of chromosome 17 (3/8) (3/10 11q23-25)	Karyotyping/FISH
Reddy, K.S. et al., Cancer Genet Cytogenet, 2000	AML/MDS	4/4	Yes (4/4)	Not Detected	Loss of chromosome 17 (2/4)	Karyotyping/FISH
Reddy, K.S. et al., Cancer Genet Cytogenet, 2001	Diffuse large B-cell lymphoma	1/1	Yes (1/1)	Not Detected	Loss of chromosome 17p (1/1)	Karyotyping/FISH
Smith, A., Cancer Genet Cytogenet, 2001	AML	1/1	Yes (1/1)	Not Detected	Loss of chromosome 17 (1/1)	Karyotyping/FISH
Starostik, P. et al., Blood, 2000	High-grade B-cell lymphoma	5/31	Yes (3/5)	Not Detected	Mutated (4/5)	FISH/CGH/Microsatellite analysis
Streubel, B. et al., Genes Chromosomes Cancer, 2000	AML/MDS	5/5	Yes (5/5)	Not Detected	Loss of chromosome 17 (1/5)	Karyotyping/FISH
Tanaka, K. et al., Blood, 1997	AML	1/6	Yes (1/1)	Not Detected	Loss of chromosome 17 (1/1)	Karyotyping/FISH
Tang, G. et al., Human Pathol, 2015	AML/MDS	21/21	Yes (21/21)	Not Detected	Mutated and/or loss of chromosome 17 (15/21)	Karyotyping/FISH/Sequencing
Tosi, S. et al., Genes Chromosomes Cancer, 1999	AML	1/1	Yes (1/1)	Not Detected	Loss of chromosome 17 (1/1)	Karyotyping/FISH/CGH
Van Limbergen, H. et al., Genes Chromosomes and Cancer, 2002	AML/MDS	6/36	Yes (6/6)	Not Detected	Loss of chromosome 17 (1/6)	Karyotyping/FISH
Zatkova, A. et al., Genes Chromosomes Cancer, 2004	AML/MDS	13/13	Yes (13/13)	Not Detected	Loss of chromosome 17 (3/13)	Karyotyping/FISH/CGH/RLGS/RT-PCR/SQ-PCR
Zatkova, A. et al., Genes Chromosomes Cancer, 2006	AML/MDS	31/31	Yes (31/31)	Not Detected	Loss of chromosome 17 (7/31)	Karyotyping/FISH/qRT-PCR/SQ-PR
Zatkova A. et al. Genes Chromosomes Cancer, 2009 (in our study)	AML/MDS	19/19	Yes (19/19)	Not Detected	Mutated (15/16)	Karyotyping/FISH/CGH/Microarray expression
Cuthbert, G. et al., Leukemia, 2000	AML/cALL	12/12	Yes (12/12)	Rearranged (3/10)	Loss of chromosome 17 (3/12)	Karyotyping/FISH/Southern Blot
Michaux, L. et al., Genes Chromosomes Cancer, 2000	AML/MDS	14/14	Yes (14/14)	Rearranged (2/7)	Loss of chromosome 17 (4/14)	Karyotyping/FISH/Southern Blot
Sarova, I. et al., Cancer Genet Cytogenet, 2010	AML	10/119 (11/partial 11q trisomy (5/10))	Yes (10/10)	Translocation (1/10)	Mutated or loss of chromosome 17 (4/10) (11q partial trisomy (2/2))	Karyotyping/FISH
Sarova, I. et al., Genes Chromosomes and Cancer, 2013	AML	20/300 (11/partial 11q trisomy (12/20))	Yes (20/20)	Rearranged (3/20) (11q partial trisomy (1/3))	Loss of chromosome 17 (7/20) (11/11q partial trisomy (4/7))	Karyotyping/FISH/BAC-based array/SNP-array
Crossen, P.E. et al., Cancer Genet Cytogenet, 1999	AML	1/1	No	Not Detected	Loss of chromosome 17 (1/1)	Karyotyping/FISH/Southern Blot/CGH
Nacheva, E. et al., Genes Chromosomes Cancer, 1993	AML	1/1	No	Not Detected	Loss of chromosome 17 (1/1)	Karyotyping/FISH
Sait, S.N.J. et al., Genes Chromosomes Cancer, 2002	AML/MDS	1/9	No	Not Detected	Loss of chromosome 17 (1/1)	Karyotyping/FISH
Cancer Genome Atlas Research et al., NEJM, 2013 (in our study)	AML	10/187	Yes (9/10)	Fusion (1/10)	Mutated (5/10)	Karyotyping/CNA/RNA-seq
Rücker, F.G. et al, Blood, 2012	AML	82/234 (11/partial 11q trisomy 60/82)	ND	ND	Mutated (76/82) (11/partial 11q trisomy (54/76))	Karyotyping/FISH/CGH/SNP-array/Sequencing
Martinez-Ramirez, A. et al., Cancer Genet Cytogenet, 2004	MDS	2/23	Yes (1/2)	Not Detected	Germline (2/2)	Karyotyping/FISH/Exon-specific PCR
Bae, S.Y. et al., Leuk & Lymphoma, 2008	AML	1/1	Yes (1/1)	Not Detected	No loss of chromosome 17 (1/1)	Karyotyping/FISH
Brezinova, J. et al., Leuk Lymphoma, 2002	AML	1/1	Yes (1/1)	Not Detected	No loss of chromosome 17 (1/1)	Karyotyping/FISH
Dolan, M. et al., Cancer Genet Cytogenet, 2001	AML/MDS	2/2	Yes (2/2)	Not Detected	No loss of chromosome 17 (2/2)	Karyotyping/FISH/Southern Blot
Haltrich, I. et al., Pathol Oncol Res, 2012	T-ALL	1/6	Yes (1/1)	Not Detected	No loss of chromosome 17 (1/1)	Karyotyping/FISH
Herry, A. et al., Ann Hematol, 2006	AML	1/1	Yes (1/1)	Not Detected	No loss of chromosome 17 (1/1)	Karyotyping/FISH
Jiang, J-G. et al., Leuk & Lymphoma, 2018	AML	1/1	Yes (1/1)	Not Detected	No loss of chromosome 17 (1/1)	Karyotyping/FISH
Kakazu, N. et al., Genes Chromosomes Cancer, 1999	MDS	1/20	Yes (1/1)	Not Detected	No loss of chromosome 17 (1/1)	Karyotyping/FISH
Lee, A.S. et al., Int J Mol Med, 2004	AML	1/1	Yes (1/1)	Not Detected	No loss of chromosome 17 (1/1)	Karyotyping/FISH
Okumura, A. et al., Tenri Medical Bulletin, 2014	AML	1/1	Yes (1/1)	Not Detected	No loss of chromosome 17 (1/1)	Karyotyping/FISH
Racke, F. et al., Cancer Genet Cytogenet, 2012	pro-B ALL	2/2	Yes (2/2)	Not Detected	No loss of chromosome 17 (2/2)	Karyotyping/FISH
Tanaka, K. et al., Leuk Lymphoma, 1998	AML/NHL/ATL	8/34	Yes (8/8)	Not Detected	ND	Karyotyping/FISH/CGH
Van Mater, D. et al., J Pediatr Hematol Oncol, 2012	B-LBL	1/1	Yes (1/1)	Not Detected	No loss of chromosome 17 (1/1)	Karyotyping/FISH
Vazquez, I. et al., Leukemia, 2004	AML	1/1	Yes (1/1)	Not Detected	No loss of chromosome 17 (1/1)	Karyotyping/FISH/RT-PCR
Wang, J. et al., Appl Immunohistochem Mol Morphol, 2012	T-ALL/LBL	14/50	Yes (14/14)	Not Detected	ND	FISH
Grygalewicz, B. et al., Am J Clin Pathol, 2018	Burkitt Lymphoma/High-grade B-cell lymphoma	11/11	Yes (9/11)	Not Detected	No loss of chromosome 17 (11/11)	Karyotyping/FISH/SNP-array/CGH
Ferreiro, J.F. et al, Haematologica, 2015	Burkitt Lymphoma	3/11	ND	ND	No loss of chromosome 17 (2/3) and ND (1/3)	Karyotyping/FISH/CGH
Rovigatti, U. et al, Science, 1986	AML/CML	2/2	ND	ND	ND	Karyotyping/Southern Blot
Salaverria, I. et al., Blood, 2014	Burkitt Lymphoma/High-grade B-cell lymphoma	13/14	ND	ND	No loss of chromosome 17 (10/13) and ND (3/13)	Karyotyping/CGH/SNP-array
Yoshida, T. et al., Cancer Genet Cytogenet, 1999	AML	1/1	ND	ND	No loss of chromosome 17 (1/1)	Karyotyping/FISH/Southern Blot
Ariyama, Y. et al., Genes Chromosomes Cancer, 1998	AML	1/1	Yes (1/1)	PTD (1/1)	No loss of chromosome 17 (1/1)	Karyotyping/FISH/nested RT-PCR
Oliveira, F.M. et al., Cancer Genet Cytogenet, 2009	AML	1/1	Yes (1/1)	Translocation (1/1)	No loss of chromosome 17 (1/1)	Karyotyping/FISH
Pajuelo-Gàmez, J.C. et al., Cancer Genet Cytogenet, 2006	AML	2/2	Yes (2/2)	PTD (2/2)	No loss of chromosome 17 (1/1)	Karyotyping/FISH
Tyybäkinoja A. et al., Genes Chromosomes Cancer, 2006	AML	2/2	Yes (1/2)	Rearranged (1/2)	No loss of chromosome 17 (1/1)	Karyotyping/FISH/CGH

11q23-25 amplified cases**			
Number of cases	*MLL* status<br>Amplification	Rearrangement	Altered *TP53*
339	329 (97%)	15 (4%)	136 (41%)

Table S6. ***TP53*** **is frequently altered in human hematologic malignancies containing 11q23-25 amplifications and lacking typical** ***MLL*** **rearrangements.** Related to Figure 4. Abbreviations: AML, Acute myeloid leukemia; MDS, Myelodysplastic syndrome; cALL, common Acute lymphoblastic leukemia; CML, Chronic myelogenous leukemia; NHL, Non-Hodgkin lymphoma; ATL, Adult T cell leukemia; LBL, Lymphoblastic lymphoma; ND, Not Determined; PTD, Partial tandem duplication. *"Loss of chromosome 17/17p" includes whole or partial loss of one or both chromosomes 17/17p. "Mutated" indicates nonsynonymous mutations and/or deletions within *TP53*. **Only cases for which we retrieved information on the *MLL* and *TP53* gene status are included.
